# Highly sensitive single-cell chromatin accessibility assay and transcriptome coassay with METATAC

**DOI:** 10.1073/pnas.2206450119

**Published:** 2022-09-26

**Authors:** Honggui Wu, Xiang Li, Fanchong Jian, Ayijiang Yisimayi, Yinghui Zheng, Longzhi Tan, Dong Xing, X. Sunney Xie

**Affiliations:** ^a^Biomedical Pioneering Innovation Center, Peking University, Beijing 100871, China;; ^b^Beijing Advanced Innovation Center for Genomics, Peking University, Beijing 100871, China;; ^c^School of Life Sciences, Peking University, Beijing 100871, China;; ^d^Peking-Tsinghua Center for Life Sciences, Academy for Advanced Interdisciplinary Studies, Peking University, Beijing 100871, China;; ^e^College of Chemistry and Molecular Engineering, Peking University, Beijing 100871, China;; ^f^Department of Bioengineering, Stanford University, Stanford, CA 94305

**Keywords:** scATAC-seq, coaccessibility, imprinted gene, joint ATAC–RNA

## Abstract

Recent advances in single-cell assay for transposase accessible chromatin using sequencing (scATAC-seq) and its coassays have transformed the field of single-cell epigenomics and transcriptomics. However, the low detection efficiency of current methods has limited our understanding of the true complexity of chromatin accessibility and its relationship with gene expression in single cells. Here, we report a high-sensitivity scATAC-seq method, termed multiplexed end-tagging amplification of transposase accessible chromatin (METATAC), which detects a large number of accessible sites per cell and is compatible with automation. Our high detectability and statistical framework allowed precise linking of enhancers to promoters without merging single cells. We systematically investigated allele-specific accessibility in the mouse cerebral cortex, revealing allele-specific accessibility of promotors of certain imprinted genes but biallelic accessibility of their enhancers. Finally, we combined METATAC with our high-sensitivity single-cell RNA sequencing (scRNA-seq) method, multiple annealing and looping based amplification cycles for digital transcriptomics (MALBAC-DT), to develop a joint ATAC–RNA assay, termed METATAC and MALBAC-DT coassay by sequencing (M2C-seq). M2C-seq achieved significant improvements for both ATAC and RNA compared with previous methods, with consistent performance across cell lines and early mouse embryos.

## Significance

The thriving field of single-cell genomics allows researchers to dissect the complexity and heterogeneity of tissues at single-cell resolution at large scale, involving transcriptome and epigenome. However, single-cell chromatin accessibility profiling methods exhibit low sensitivity. Here, we increased accessible chromatin detection sensitivity in single cells with METATAC, a single-cell ATAC-seq technique, with the help of META amplification strategy and other biochemical modifications. METATAC reached the highest accessible chromatin region detection efficiency compared with existing techniques, allowing more accurate cis-regulatory element coaccessibility measurement and allele-specific chromatin accessibility analysis in complex tissue samples. In combination with a high-resolution single-cell RNA sequencing assay, we further developed a high-sensitivity joint single-cell ATAC–RNA strategy, which helps us to better resolve gene regulatory programs.

Eukaryotic DNA is wrapped around a core of histone proteins (1), hierarchically organized into chromatin and having limited accessibility to external factors. Chromatin accessibility to regulatory factors is dynamically regulated in a cell type–specific manner (2, 3). Various genome-wide methods have been developed to identify active regulatory regions and transcription factor (TF) footprints (4–7). However, these methods measure ensemble chromatin states from a population of cells, omitting the heterogeneity among cells. The development of single-cell chromatin accessibility assays (e.g., single-cell assay for transposase accessible chromatin using sequencing [scATAC-seq]) enabled the capture of accessible chromatin regions at single-cell resolution. However, current scATAC-seq methods have low detection efficiency (8–15), detecting a limited number of accessible regions from each cell. Therefore, it is often necessary to average over many chromatin regions [e.g., regions with the same TF binding motifs or all peaks from a chromatin immunoprecipitation followed by sequencing (ChIP-seq) experiment (16)] and/or merge many single cells for downstream analysis (10). The low sensitivity of these methods also presents a major hurdle for understanding gene regulation mechanisms through single-cell multimodal coassays. Simultaneous measurement of chromatin accessibility and gene expression from the same cells could in principle assign cis-regulatory elements (CREs) to target genes and identify primed chromatin states foreshadowing gene expression. However, existing single-cell ATAC-RNA coassays have low sensitivity (17–22), restricting their ability to recover biologically important correlations between chromatin accessibility and gene expression.

Here we present multiplexed end-tagging amplification of transposase accessible chromatin (METATAC), a plate-based, automation-compatible, high-sensitivity scATAC-seq method and its coassay with simultaneous single-cell RNA sequencing (scRNA-seq) measurements. Through extensive biochemical optimization, METATAC exhibits dramatically improved sensitivity in detecting accessible chromatin regions from single cells. We further developed a coaccessibility metric without merging single-cell data, which precisely links enhancers to promoters and recapitulates long-range interactions between CREs. Applying METATAC to the cerebral cortex of an F1 hybrid mouse, we identified thousands of cell type–specific, monoallelically accessible sites and studied the allele-specific accessibility state of imprinted genes. Finally, we combined METATAC with our scRNA-seq method multiple annealing and looping based amplification cycles for digital transcriptomics (MALBAC-DT) (23), which offers high mRNA capture efficiency and accurate unique molecular identifier (UMI) counting, to develop a sensitive single-cell joint ATAC-RNA method—METATAC and MALBAC-DT coassay by sequencing (M2C-seq). This coassay consistently outperformed most previous methods on different cell lines. We further applied M2C-seq to early mouse embryos and recapitulated the differentiation trajectories of embryonic and extraembryonic lineages.

## Results

### Highly Sensitive scATAC-seq with METATAC.

**Fig. 1.**
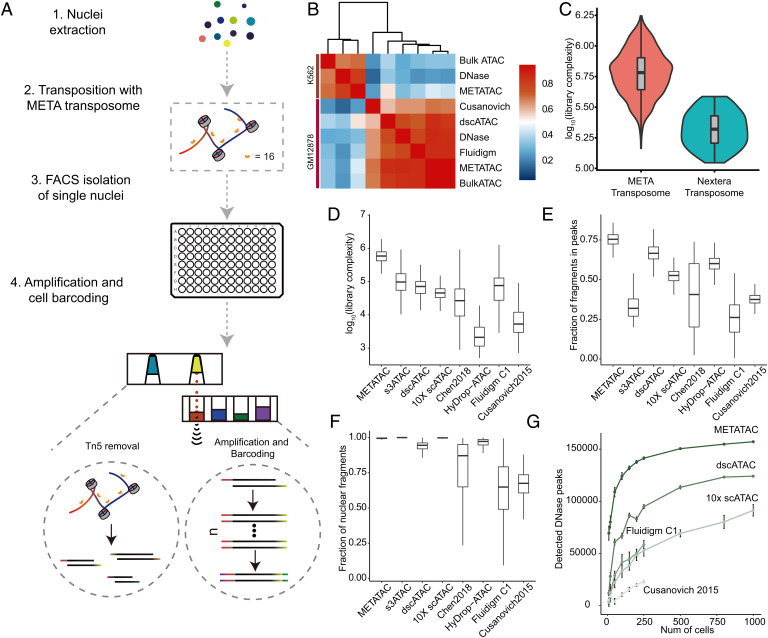
METATAC shows high detection efficiency in single cells. (*A*) Schematic workflow of METATAC. Cells are permeabilized with Omni-ATAC lysis; then nuclei are transposed with META transposome, single nuclei are sorted to 96-well plates via flow cytometry, Tn5 is removed with SDS from bound DNA, and META amplification and cell barcoding are conducted in two steps via an acoustic liquid transfer system. (*B*) Spearman correlation of chromatin accessibility peaks across bulk and single-cell datasets for K562 and GM12878. (*C*) Comparison of library size between META transposome (*n* = 1,099) and conventional Nextera transposome (*n* = 87) using GM12878 cells. Two samples are sequenced at the same depth, and library size was estimated with the Lander–Waterman equation. QC metrics of scATAC-seq technologies: (*D*) Library size of all technologies. The median library size for METATAC is 587,707 reads (*n* = 1,099, GM12878), as compared with s3-ATAC (26) (97,142 reads, *n* = 2,174, human prefrontal cortex), 10× scATAC-seq (13) (45,515 reads, *n* = 5,297, GM12878), dscATAC (12) (66,618 reads, *n* = 2,295, GM12878), Chen plate-based scATAC-seq (14) (26,700 reads, *n* = 384, K562), HyDrop-ATAC (27) (2,114 reads, *n* = 1,141, MCF-7), Fluidigm C1 (8) (33,529 reads, *n* = 382, GM12878), and sciATAC-seq methods (9) (5,300 reads, *n* = 533, GM12878). (*E*) Fraction of reads mapped in peaks. For this comparison, peaks are called for each technology individually. The median FRiP of METATAC is 75.412%. (*F*) Proportion of reads mapped to nuclear genome. The median for METATAC is 95.552%. (*G*) Comparison of detected DHSs versus cell number across different techniques of GM12878. Each cell number is randomly performed three times.

In METATAC, nuclei are extracted with an improved permeabilization procedure, followed by bulk transposition with our META transposome (24). Transposed nuclei are then isolated via fluorescence-activated cell sorting into 96-well plates, where Tn5 release, DNA amplification, and cell barcoding are performed. All steps are robotically automated with a pipette-free, acoustic liquid transfer system, which can simultaneously profile 2,000 cells (Fig. 1*A*).

We increased sensitivity from three aspects. First, we adopted the permeabilization step from Omni-ATAC (25), which significantly reduced backgrounds and mitochondrial reads fraction and has been independently adopted by another method (12). Second, we used our META transposome instead of the conventional (Nextera) Tn5 transposome in the transposition step, which avoids a 50% loss of starting fragments caused by self-looping in the traditional transposon design (24). Third, we used an ionic detergent, sodium dodecyl sulfate (SDS), to remove Tn5 from bound chromatin before amplification to maximize DNA recovery.

We performed a proof-of-concept experiment on two well-characterized human cell lines, GM12878 (lymphoblastoid) and K562 (leukemia), profiling 1,224 and 768 cells, respectively; after rigorous quality control (QC) (*Materials and Methods*), we finally got 1,099 (89.8%) GM12878 cells and 747 (97.2%) K562 cells, each of which contained 142,280 and 206,435 unique fragments (median), respectively, at current sequencing depth (479,951 reads and 724,412 reads per cell, respectively [median], nearly saturated). We compared our averaged data with published datasets and found the highest correlation to be between METATAC and bulk DNase-seq data from respective cell lines (Fig. 1*B*). To demonstrate the advantage of using META transposome, we performed a side-by-side comparison on GM12878 cells and found the library complexity generated by the META transposome is much higher than the Nextera transposome (median number of unique fragment per cell = 142,280 versus 98,123) (Fig. 1*C*). Using GM12878 for benchmarking, we found METATAC to have a 9- to 100-fold increase in library complexity (Fig. 1*D*) compared with the previous methods (8, 9, 12–14, 26, 27). Besides, our data show negligible mitochondrial reads (less than 0.5%) and significant enrichment for fragments in accessible chromatin regions (75% fraction of reads in peaks [FRiP]) (Fig. 1 *E* and *F*). We detected only 1 doublet in 345 high-quality nuclei (0.3%; *SI Appendix*, Fig. S1*A*) from a 1:1 mixture of human and mouse cells, suggesting a much lower collision rate compared with other methods (9, 12, 13). METATAC also showed significant enrichment for fragments within transcription start site (TSS) regions and a fragment-size distribution similar to other methods (*SI Appendix*, Fig. S1 *B* and *C*). For counting unique fragments detected in single cells, specifically, to avoid potential cell-to-cell contamination due to cell barcode switching during library preparation or index hopping during sequencing, we introduced a stringent decontamination procedure, which only kept the fragment in cells with the highest frequency (*Materials and Methods*).

To demonstrate METATAC’s sensitivity in detecting DNaseI hypersensitive sites (DHSs), a gold standard for accessible chromatin regions in bulk cells, we averaged our data from different numbers of cells. Here we defined that if peaks called from merged cells overlapped with DHSs, then the DHS is considered as detected. Compared with other techniques, METATAC detected many more DHSs at small cell numbers (median 71,600 from 10 cells, median 81,500 from 20 cells) and detected an additional ∼26,000 DHSs even when other techniques are saturated (Fig. 1*G*). We found our data to have a higher fraction of reads in DHSs (48.46%) compared with other methods (*SI Appendix*, Fig. S1*E*). Aggregating chromatin accessibility profiles at different cell numbers showed that METATAC detects 45.6% of total DHSs even with as few as 10 cells (*SI Appendix*, Fig. S1*F*), suggesting high sensitivity and low background.

Altogether, these improvements combined with an acoustic liquid transfer system make METATAC a high-efficiency, high-quality, and high-throughput single-cell epigenome profiling method.

### High-Sensitivity Data Revealed Coaccessibility of Functionally Related Regulatory Elements.

**Fig. 2.**
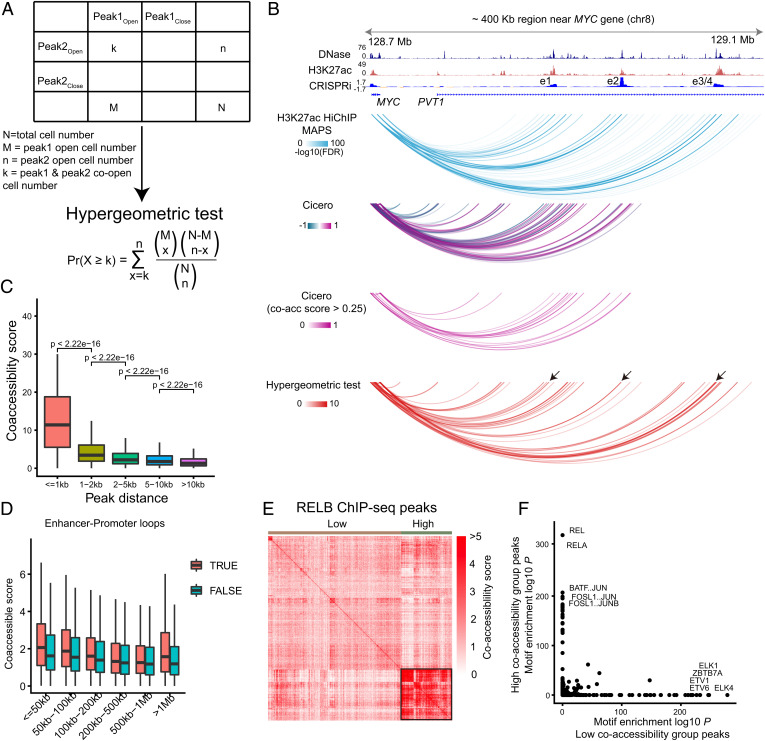
High-detectability METATAC revealed the coactuation of functionally related regulatory elements. (*A*) Definition of coactuation in METATAC with hypergeometric test. (*B*) METATAC coaccessibility recovers independently validated long-range interactions. The coaccessibility score was calculated with K562 cells (*n* = 747). A CRISPRi screen for MYC locus on K562 identified seven distal enhancer regions (29), four of which are shown here. Model-based Analysis of PLAC-seq and HiChIP (MAPS) for K562 H3K27ac HiChIP data, Cicero calculated coaccessibility (all and threshold >0.25 are shown), and hypergeometric test calculated coaccessibility (score >2 are shown) only show MYC anchored links. CRISPRi identified enhancers are highlighted with arrowheads. (*C*) Coaccessibility score of neighboring peaks as a function of the distance between the accessible peaks, which was calculated with GM12878 cells (*n* = 1,099). (*D*) Coaccessibility score of E–P loop anchors, grouped by loop size, which was calculated with GM12878 cells (*n* = 1,099). E–P loops are called from in situ Hi-C data at 5-kb resolution (32). True represents E–P loop anchors, and false represents regions neighboring loop anchors. (*E*) Hierarchical clustering of RELB ChIP-seq peaks based on coaccessibility scores. Two groups were identified, which we named the high-coaccessibility group and the low-coaccessibility group according to coaccessibility scores within each group, which are calculated with GM12878 cells (*n* = 1,099). The high-coaccessibility group is highlighted. For visualization, only 2,000 peaks were subsampled. (*F*) TF motif enrichment for the high-coaccessibility group and the low-coaccessibility group in *E*.

Previous coaccessibility algorithms need to merge multiple cells to alleviate data sparsity, which may introduce false positives and false negatives due to cellular heterogeneity (10). Here, using our high-detectability data, we developed a statistical strategy to quantify coaccessibility between two accessible chromatin regions, which is not limited by their separating genomic distance and does not merge single cells. First, we called 93,155 accessible chromatin regions (peaks) from GM12878 with a median of 31,378 peaks detected in each cell. We then implemented a hypergeometric test for each pair of peaks, taking individual peak accessibility into account (Fig. 2*A*). With this metric, we investigated coaccessibility between neighboring peaks as a function of linear distance with GM12878 cells (*n* = 1,099), which decreases very quickly within 1 kb (Fig. 2*B*), concordant with long-read single-molecule sequencing data (28).

Next, we compared our coaccessibility metric with a popular coaccessibility algorithm Cicero (10), using the MYC locus as an example because it has independent CRISPRi validation results (29). We found our coaccessibility links (calculated with K562 cells, *n* = 747) to precisely recover validated long-range interactions (Fig. 2*C*), including four enhancers (e1 to e4) identified by CRISPR interference (CRISPRi), while Cicero missed the most distal two (e3 and e4). To systematically compare the performance of the hypergeometric test and Cicero, we used another CRISPRi dataset (30) to calculate the precision-recall statistics. We found that the hypergeometric test outperforms Cicero (Area Under Curve [AUC] = 0.697 versus 0.625) for enhancer–promoter (E–P) pairs within 10 kb; for E–P pairs >10 kb, Cicero exhibits a slightly better result (AUC = 0.084 versus 0.043) (*SI Appendix*, Fig. S2*D*). Indeed, validated E–P pairs have higher coaccessibility scores (*SI Appendix*, Fig. S2*B*). These results indicate coaccessibility alone has weak prediction ability for true E–P pairs; it would be better to incorporate other experimental datasets (e.g., High-throughput Chromosome Conformation Capture (Hi-C), Chromatin Interaction Analysis with Paired-End Tag (ChIA-PET), Proximity Ligation-Assisted ChIP-Seq (PLAC-seq), HiChIP, and CRISPRi).

Besides, our hypergeometric test enables custom peak sets (including long-range peak pairs and interchromosomal peak pairs), but Cicero only restricts to promoter-centered peak sets. Benefiting from this, we found our coaccessibility metric to recapitulate megabase-scale long-range interactions, for example, in the HIST1 clusters that span 2 Mb (*SI Appendix*, Fig. S2*C*). These results indicate that our hypergeometric test is well suited for coaccessibility analysis.

Enhancers are known to activate target genes by forming physical interactions with promoters via bound TFs and cofactors (31); however, whether E–P loop anchors tend to be coaccessible is unknown. We inspected their coaccessibility using E–P loops called from high-resolution in situ Hi-C data (32) and found loop anchors are more likely to be coaccessible compared with neighboring nonloop anchor peaks (calculated with GM12878 cells, *n* = 1,099), which is not dependent on linear distance (Fig. 2*D*). All these results indicate that our coaccessibility score accurately reflects enhancer regulatory potential on target genes and recovers long-range three-dimensional genome architecture.

It remains unknown whether TF binding sites, which are scattered across different chromosomes, show coaccessibility. To explore this property, we calculated coaccessibility scores for ChIP-seq peaks of a subunit of NFκB, RELB, in GM12878. We found RELB binding sites cluster as two groups. One group exhibited a high-coaccessibility pattern (Fig. 2*E*), while the other did not. To explain this phenomenon, we conducted motif enrichment within the two groups. To our surprise, we found that the NFκB motif was enriched only in the high-coaccessibility group (Fig. 2*F*). ChIP-seq peaks have false positives due to indirect TF binding and nonspecific binding. Binding sites that showed high coaccessibility might indicate direct TF binding, and sites without significant coaccessibility may be derived from indirect TF binding or nonspecific binding. This needs experiments and further analysis to validate.

### The Epigenomic Landscape of Adult Mouse Cerebral Cortex.

**Fig. 3.**
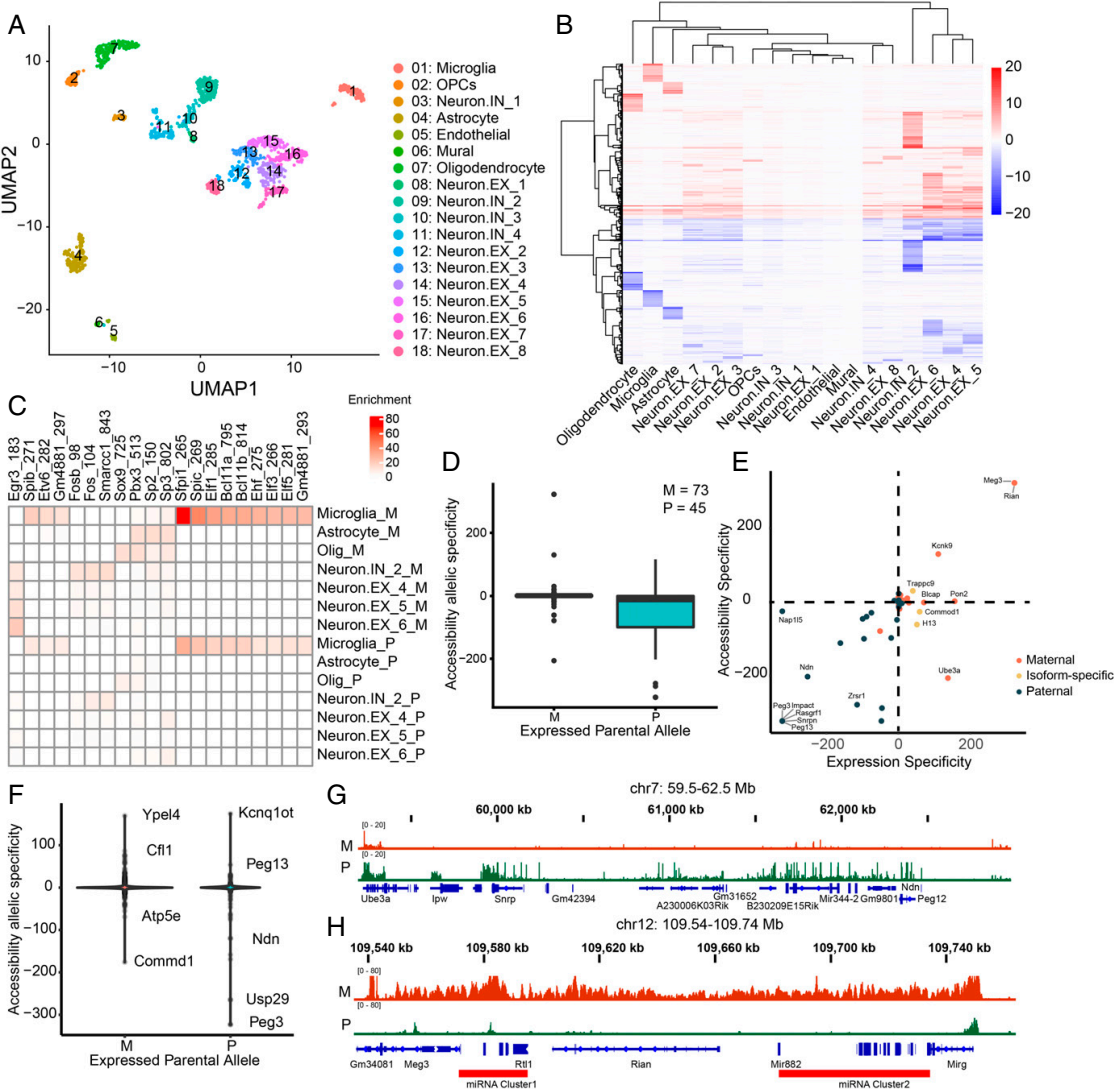
Allele-specific accessibility landscape of mouse brain. (*A*) Uniform Manifold Approximation and Projection (UMAP) visualization of cells derived from mouse cerebral cortex (*n* = 1,370). (*B*) Allele-specific accessibility of cell types in *A*. Red indicates maternal-specific, and blue indicates paternal-specific. (*C*) TFs motif enrichment for allele-specifically accessible peaks. (*D*) Allele-specific accessibility of imprinted genes, grouped as maternally imprinted genes and paternally imprinted genes. Positive value indicates maternal-specific accessibility, and negative value indicates paternal-specific accessibility. (*E*) Scatterplot between expression specificity and accessibility specificity of imprinted genes. (*F*) The accessibility specificity of enhancers linked to imprinted genes, grouped by maternal/paternal imprinting. (*G*) A paternal-specifically accessible region (chr7, 59.5 to 62.5 Mb). (*H*) A maternal-specifically accessible region (chr12, 109.54 to 109.74 Mb). Two microRNA clusters are labeled.

To further demonstrate the superiority of METATAC, we profiled accessible chromatin in 1,536 cells (after filtering, 1,370 cells retained) from the adult mouse cerebral cortex. After cell clustering, we identified 18 clusters, capturing all main cell types, including astrocytes, microglia, oligodendrocytes, oligodendrocyte progenitor cells, mural cells, endothelial cells, eight types of excitatory neurons, and four types of inhibitory neurons (Fig. 3*A*). After rigorous filtering, the median number of unique fragments per cell is 66,064. The fragment length distribution and TSS enrichment indicate the high quality of the dataset (*SI Appendix*, Fig. S3 *A* and *B*). We faithfully conducted TF footprint analysis with high detectability even with the modest cell count (*SI Appendix*, Fig. S3*C*). Correlation with a published scRNA-seq dataset (33) (*SI Appendix*, Fig. S3*D*) and marker gene activity projection further validated our cell type annotation (*SI Appendix*, Fig. S3 *E*–*J*).

Using the resulting data, we aggregated the METATAC profiles in each cluster and determined the accessible chromatin regions with ArchR (34). We identified 197,464 candidate CREs (cCREs) in total, overlapping with 74.62% DHSs mapped in bulk mouse cortex tissues by the Encyclopedia of DNA Elements (ENCODE) consortium (35) (*SI Appendix*, Fig. S4*A*).

For further analysis, we identified 161,390 cell-type-specific cCREs (*SI Appendix*, Fig. S4*B*). Calculating the deviation of TF activities across clusters with chromVAR (16), we could determine cell type–specific TF regulators for each cluster (*SI Appendix*, Fig. S4*C*). Many of them overlapped with known master regulators for each cell identity. Among the identified TFs, we found FOS motif activity shows variability within neuron clusters, reflecting neural activity (12, 36), with one cluster of inhibitory neurons IN_2 showing much higher Fos activity than other inhibitory neurons (*SI Appendix*, Fig. S4*D*). Neurons EX.4 and EX.6 were enriched with NEUROG11, NEUROD1, and NEUROD2, which are essential TFs for the development of the cerebral cortex. These known key TFs (*SI Appendix*, Fig. S4 *E*–*G*) suggest that our methods are sufficient to study gene regulation programs for various lineage with a relatively modest cell number.

### Systematic Interrogation of Allele-Specific Accessibility in Mouse Brain.

Because the cerebral cortex we used is from F1 mouse hybrids of CAST/EiJ and C57BL/6J cross, we sought to study allele-specific accessibility landscape in the mouse cerebral cortex (37). Within the defined cCREs, we identified 10,243 allele-specifically accessible peaks in total, among which 48.5% are paternally accessible, and the remaining peaks are maternally accessible (Fig. 3*B*). Interestingly, most monoallelically accessible peaks are cell type–specific peaks as well (*SI Appendix*, Fig. S5*A*). Among all the monoallelically accessible peaks, 39.1% are distal elements, 7.6% are promoters, 4.5% are located at exons, and the remaining overlap with intronic regions. We then checked the TF motif enrichment in these monoallelically accessible sites; we identified EGR3 specifically enriched in maternally accessible peaks of most neuron types (Fig. 3*C*). Egr3 shows corresponding activity in neurons (*SI Appendix*, Fig. S5*B*), indicating that EGR3 may play an essential role in regulating neuronal monoallelic accessibility, but we could not exclude the strain-specific binding difference of EGR3.

Next, we were curious whether imprinted genes show monoallelic accessibility. We calculated the allele specificity of accessibility at the promoter region of all known imprinted genes (binomial test; *Materials and Methods*). Surprisingly, we found most maternally imprinted genes show no significant accessibility difference between the two alleles, while most paternally imprinted genes show paternally specific accessibility (Fig. 3*D* and *SI Appendix*, Fig. S5 *E* and *F*). We examined the accessibility profile in each cell type and found that this pattern was consistent across cell types. We then examined whether maternally imprinted genes had lower accessibility, but we found accessibility specificity did not correlate with accessibility level (*SI Appendix*, Fig. S5*C*). In order to focus on imprinted genes that are expressed in adult mouse cerebral cortex, we performed a bulk RNA-seq on the mouse cerebral cortex from mice the same as used for METATAC. We detected 38 maternally imprinted genes and 25 paternally imprinted genes in the bulk RNA-seq dataset. Of these, 29 maternally imprinted genes have <80% transcripts originating from the maternal allele, among which 18 have <60% (*SI Appendix*, Fig. S5*D*), while 70% of paternally imprinted genes have >90% transcripts originating from the paternal allele, indicating most of the maternally imprinted genes exhibit maternally biased expression and the majority of paternally imprinted genes show almost monoallelic expression. We then plotted promoter accessibility specificity versus expression specificity of imprinted genes (binomial test; *Materials and Methods*). We found that paternally imprinted genes show a high correlation but not maternally imprinted genes (Fig. 3*E*). Taken together, we found many maternally imprinted genes show biallelic accessibility; we speculated they may adopt a posttranscriptional mechanism to achieve maternally biased expression, such as previously reported miRNA-mediated paternal mRNA degradation (38). For paternally imprinted genes, we could not rule out the possibility that the observed monoallelic accessibility is derived from strain-specific accessibility. We found isoform-specific imprinting genes show no accessibility difference at gene promoter for both alleles but show allele-specific accessibility at specific gene body regions (*SI Appendix*, Fig. S5*H*). Inspecting all genes did not show a strong correlation between accessibility specificity and expression specificity, and many genes with maternally biased expression showed no allele-specific accessibility (*SI Appendix*, Fig. S5*G*).

Next, we are curious about whether enhancers regulating imprinted genes have allele-specific accessibility. We first connected putative enhancers to imprinted genes by measuring the coaccessibility, then calculated the accessibility allelic specificity. Interestingly, we found almost all enhancers showed biallelic accessibility, no matter whether they are regulating maternally or paternally imprinted genes (Fig. 3*F* and *SI Appendix*, Fig. S5*I*), except for those genes linked to other imprinted genes (e.g., Ndn, Peg3, and Ypel4). Among the monoallelically accessible sites, most of their flanking peaks were biallelically accessible with two notable exceptions. One is Prader–Willi syndrome and Angelman syndrome (PWS/AS) locus on chr7 (chr7, 59.5 to 62.5 Mb), a 3-Mb region that shows paternal-specific accessibility (Fig. 3*G*), which encompasses 20 paternally imprinted genes. Another is a 200-Kb region on chromosome 12 (chr12, 109.54 to 109.74 Mb) that shows maternal-specific accessibility (Fig. 3*H*), including seven maternally imprinted genes (e.g., Meg3, Rian, and Mirg) and two known imprinted microRNA clusters (39).

### Construct In Vivo Active/Inactive X Chromosome Accessibility Landscape.

It is challenging to study the allele-specific accessibility of chromosome X in vivo due to random paternal/maternal X chromosome inactivation. To construct the active and inactive X chromosome accessibility landscape of in vivo mouse brain, we first determined which X chromosome was inactivated in single cells by calculating the maternal fragment ratio in all SNP-informative fragments mapped to the X chromosome. As expected, we observed a bimodal distribution, which peaked near 0 and 1 (*SI Appendix*, Fig. S6*A*), corresponding to maternal X (Xm) and paternal X (Xp) inactivation, respectively. Xp and Xm inactivation ratio shows no significant difference among cell types (*SI Appendix*, Fig. S6*B*), and Xp/Xm inactivated cells mixed within the same cell type (*SI Appendix*, Fig. S6*C*). Then we merged all the fragments from the active X chromosome and inactivated X chromosome (*SI Appendix*, Fig. S6*E*). As expected, the inactive X chromosome exhibits limited accessible sites, among which most accessible sites overlap with known escapees in the brain (40) (e.g., Kdm5c, Kdm6a, Ddx3x, and Tmen29). Nonbrain escapees show nearly no accessibility in Xi (*SI Appendix*, Fig. S6*D*). We then focused on regions essential for X chromosome inactivation, the X inactivation center (Xic), and the Firre locus. Xic contains noncoding RNA Xist and its regulators. Xist is Xi-specific accessible as expected, while neighboring regulators, like Jpx and Ftx, are biallelically accessible (*SI Appendix*, Fig. S6 *E*, *Right*). Rlim shows Xa-specific accessibility in the adult mouse brain, while it is biallelically accessible in neural progenitor cells (NPC) (41) (*SI Appendix*, Fig. S6*F*), indicating X chromosome inactivation is not fully completed in NPC. Interestingly, the Firre promoter region is Xa-specific accessible, while the Firre gene body is Xi-specific (*SI Appendix*, Fig. S6 *E*, *Left*), which showed biallelic accessibility in NPC (*SI Appendix*, Fig. S6*F*).

### Development of a High-Sensitivity Single-Cell Joint ATAC-RNA Assay.

The precise spatiotemporal gene expression program is regulated by the underlying epigenomic landscape, including chromatin accessibility. To better understand the epigenomic programs regulating cell type–specific gene expression, it is vital to jointly profile gene expression and chromatin states within the same cells. Several joint ATAC–RNA assays have been developed (17–22, 42). However, all these methods show low detectability, recovering only hundreds to several thousands of fragments or RNA molecules for ATAC and RNA.

**Fig. 4.**
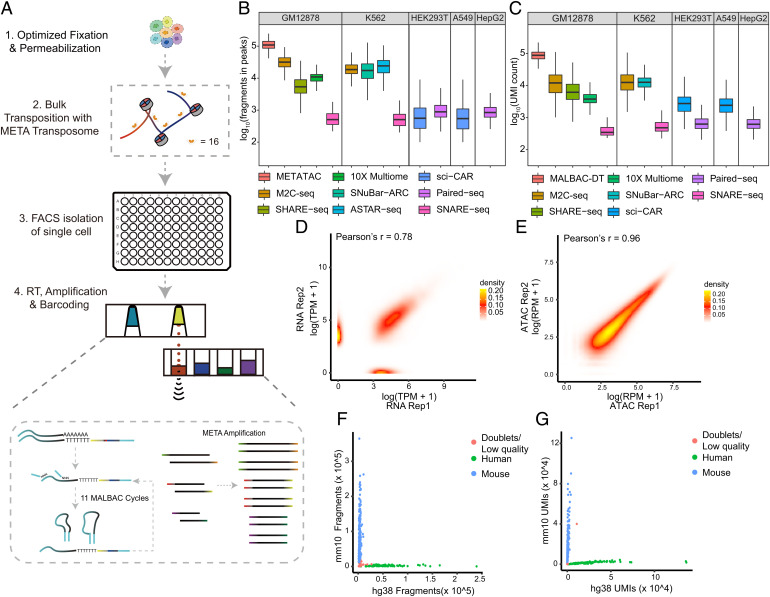
Joint single-cell transcriptome and chromatin accessibility sequencing. (*A*) Workflow of our simultaneous ATAC-RNA method. (*B*) Number of ATAC fragments in peaks. The median number of M2C-seq (GM12878, 31,790, *n* = 511; K562, 18,540, *n* = 359) is compared with METATAC only (GM12878, 108,792, *n* = 1,186), SHARE-seq (17) (GM12878, 5,363, *n* = 1,204), 10× multiome (GM12878, 10,924, *n* = 2,714), SNuBar-ARC (42) (K562, 17,369, *n* = 5,131), ASTAR-seq (21) (K562, 24,231, *n* = 136), sci-CAR (18) (HEK293T, 558, *n* = 711; A549, 545, *n* = 3,427), SNARE-seq (19) (GM12878, 507, *n* = 140; K562, 507, *n* = 200), and Paired-seq (20) (HEK293T, 885, *n* = 1,833; HepG2, 843, *n* = 1,186). (*C*) Number of RNA UMIs. The median number of M2C-seq (GM12878, 12,068, *n* = 560; K562, 12,494, *n* = 359) is compared with MALBAC-DT only (GM12878, 87,728, *n* = 948), SHARE-seq (17) (GM12878, 6,173, *n* = 1,159), 10× multiome (GM12878, 3,716, *n* = 2,714), SNuBar-ARC (K562, 12,642, *n* = 6,136), ASTAR-seq (K562, *n* = 192), sci-CAR (18) (HEK293T, 2,752, *n* = 812; A549, 2,419, *n* = 4,277), SNARE-seq (19) (GM12878, 346, *n* = 140; K562, 482, *n* = 200), and Paired-seq (20) (HEK293T, 628, *n* = 1,174; HepG2, 620, *n* = 1,141). Scatterplots show the correlation of read counts from two technical replicates of (*D*) RNA profiles and (*E*) ATAC fragment profiles. (*F*) Unique ATAC fragments and (*G*) RNA UMIs mapped to human or mouse genome. The species mixing experiment is performed on a mix of human (K562) and mouse (mESC V6.5) cell lines.

To achieve high sensitivity in both modalities, we optimized the procedure from two aspects, including optimization of the fixation condition and the combination of two high-resolution methods, METATAC and MALBAC-DT (23), and we developed M2C-seq. Briefly, fixed cells were permeabilized and bulk transposed with the META transposome and were isolated into 96-well plates via flow cytometry; then we conducted cDNA and ATAC fragment coamplification in the same well; finally, amplified products were split into two parts for ATAC and RNA enrichment. Specifically, we incorporated the amplification steps with an acoustic liquid transfer system (Fig. 4*A*).

To validate data quality, we profiled the human lymphoblastoid cell line GM12878. We generated two replicates with a total of 960 cells, of which 862 (89.8%) passed ATAC QC and 745 (77.6%) passed RNA QC, and finally retained 682 (71%) cells that passed both QCs. For GM12878, the median number of unique ATAC fragments is 88,767 (52.11% FRiP) (Fig. 4*B*); the median number of RNA UMIs is 12,068 (median 3,438 genes) (Fig. 4*C*), and both modalities outperformed most published joint ATAC–RNA methods. The fragment size distribution, enrichment at TSS, and high FRiP indicate the high quality of our data (*SI Appendix*, Fig. S7 *A*, *B*, and *H*). We noted that coassay detects fewer ATAC fragments than METATAC alone, which might arise from fixation needed to retain more RNA. However, fixation affects Tn5 transposition specificity and reduces tagmented DNA recovery.

Our method shows a high correlation between replicates (Fig. 4 *D* and *E*) and exhibits high consistency with bulk DNase and METATAC profiles (*SI Appendix*, Fig. S7*E*). In addition, our method has a similar performance on other cell lines (Fig. 4 *B* and *C* and *SI Appendix*, Fig. S7*D*). To estimate data specificity, we performed an experiment on a mixture of human (K562) and mouse (mESC V6.5) cell lines. Human and mouse reads separate well for both modalities (Fig. 4 *F* and *G*), showing low contamination. Both gene expression profile and chromatin accessibility separate GM12878 (*n* = 511) and K562 (*n* = 359) cell lines well (*SI Appendix*, Fig. S7 *F* and *G*), and differential gene expression analysis identified marker genes for each cell type (*SI Appendix*, Fig. S7*I*).

Taken together, through fixation optimization and the combination of two sensitive methods, METATAC and MALBAC-DT, we developed a single-cell high-sensitivity simultaneous ATAC-RNA assay, achieving a high detection rate of gene expression and chromatin accessibility within the same cells.

### Joint Profiling of Chromatin Accessibility and Gene Expression from Mouse Blastocyst Embryo.

Next, we profiled 512 cells from mouse E4.5 and E5.5 embryos to demonstrate M2C-seq performance on tissue samples (*SI Appendix*, Fig. S9 *A*–*D*). After rigorous QC, we retained 328 high-quality cells (E4.5, 185 cells; E5.5, 143 cells), of which cells passing QC had a median of 73,716 unique ATAC fragments and 18,070 RNA UMIs (4,523 genes) (*SI Appendix*, Fig. S8*C*).

We then clustered and annotated cell types based on known marker genes (43–45). For RNA, we got seven clusters, including three clusters of epiblasts (Epi), two types of primitive endoderm (PrE), parietal endoderm (PE), and visceral endoderm (VE) (*SI Appendix*, Fig. S8*A*). Marker gene expression confirmed our cell type annotation (*SI Appendix*, Figs. S8*B* and S9*J*). ATAC clusters show high congruence with RNA clusters (*SI Appendix*, Figs. S8*E* and S9 *E*–*H*). E4.5 epiblasts separate as two types according to the transcriptome, while their epigenome is indistinguishable (*SI Appendix*, Figs. S8*E* and S9 *G* and *H*). Primitive endoderm shows a differential transcriptional and epigenomic state at E4.5, implying future differentiation into PE and VE at E5.5, respectively. For most genes, their accessibility is highly consistent with their expression (*SI Appendix*, Figs. S8*F* and S9 *K*–*M*). In comparison, some genes’ accessibility foreshadows gene expression, indicating chromatin priming (*SI Appendix*, Fig. S8*D*). In addition, we found Dnmt3b and Tet1 expression is divergent among PE and VE lineage (*SI Appendix*, Fig. S9 *I* and *J*). Taken together, our data precisely recaptured early mouse embryonic and extraembryonic lineage development, revealing chromatin priming for lineage genes, which is predictive of cell state transition.

## Discussion

Our work presents a highly sensitive, fully automated single-cell ATAC-seq method, termed METATAC. With high detectability, we defined a coaccessibility metric without merging single cells, which could link more distal enhancers to target genes and recapitulates the three-dimensional long-range interactions between CREs. Then we systematically investigated allele-specific accessibility landscape in mouse cerebral cortex and the allele specificity of accessibility of imprinted genes. Specifically, we found many maternally imprinted genes show biallelic accessibility, while paternally imprinted genes show paternal-specific accessibility. We speculated that this is related to their different expression patterns, in which most maternally imprinted genes show maternally biased expression (except Kcnk9 and Blcap), while the majority of paternally imprinted genes show almost monoallelic expression. Thus, different mechanisms maybe are adopted to regulate their expression. Next, to demonstrate METATAC compatibility, we combined a high-sensitivity scRNA-seq method, MALBAC-DT, to develop a high-resolution single-cell joint ATAC–RNA assay, which consistently outperformed most previously published methods and shows similar performance on different cell lines and mouse early embryos.

Only a few studies addressed monoallelic accessibility, including profiles of mouse ESC-derived clonal NPCs (46) and human induced pluripotent stem cell (iPSC)-derived neurons (47), revealing that allele-specific accessible chromatin regions are enriched with neuropsychiatric disease risk variants. We profiled in vivo allele-specific accessibility in the mouse brain and revealed cell type–specific accessibility patterns, which offer a valuable resource for neuropsychiatric disease study. This needs reciprocal cross to filter out strain-specific accessible sites. We found Egr3 is enriched in neuronal maternally accessible peaks, and Egr3 is a potential susceptibility candidate in schizophrenia (48).

Regarding high-throughput single-cell ATAC-seq techniques, single-cell combinatorial indexing (sci)-based and droplet-based techniques achieve 1 × 10^3^ to 8 × 10^4^ cells per experiment, while current METATAC only profiles 2,000 cells in a single experiment, although with high sensitivity. However, the three levels of sci-ATAC-seq3 suffer from low sensitivity and poor data quality (estimated library size is 9,878, and fraction of reads in DHSs is 0.2) (49), and droplet-based scATAC-seq (e.g., 10× Chromium scATAC-seq) generates a noticeable proportion of “barcode multiplets”—a single droplet contains multiple oligonucleotide barcode sequences (50), which could confound single-cell analysis. For tissues or systems with defined cell types, thousands to tens of thousands of cells are enough to analyze the epigenetic regulation, while the cell atlas study requires single-cell sequencing at scales of hundreds of thousands to millions of cells (49, 51–53). The increasing application of single-cell omics technologies to cell atlas study prompts us to adopt sci strategy to increase throughput (9, 11, 54) in the future. In combination with combinatorial indexing, we could increase throughput to 20,000 to 50,000 cells in a single experiment without compromising data quality too much, which needs the usage of molecularly barcoded Tn5, limiting its wide adoption. Besides, we need to make a trade-off between cell number and sequencing depth when performing cell atlas study. The adoption of acoustic liquid transfer system helps us to reduce labor costs and increase throughput, but the cost of Echo liquid handler is prohibitive; we also validated our workflow on cheaper alternative liquid handlers—I-DOT and mosquito.

## Materials and Methods

### Ethics Statement.

The study was approved by the Peking University Institutional Animal Care and Use Committee. All the animal experiments were conducted following their guidelines.

### Cell Culture.

All cell lines were grown at 37 °C with 5% CO_2_ at recommended density. K562 (ATCC) chronic myeloid leukemia cells were cultured in Iscove’s Modified Dulbecco’s medium (Gibco, cat. no. 12440053) supplemented with 10% FBS (Gibco, Thermo Fisher Scientific, Cat. #10099141) and 1% Penicillin Streptomycin (Pen/Strep) (Gibco, Thermo Fisher Scientific, cat. no. 15140148). K562 cells used in this study were from a single cell clone. GM12878 (ATCC) lymphoblastoid cells were grown in Roswell Park Memorial Institute 1640 Medium (Gibco, Thermo Fisher Scientific, cat. no. 11875093) media supplemented with 15% FBS and 1% Pen/Strep. GM12878 cells were grown from a single cell clone. V6.5 mouse embryonic stem cells (kind gift from the Peng Du laboratory, Peking University, Beijing, China) were cultured in Glasgow Minimum Essential Medium (Sigma-Aldrich) supplemented with 15% FBS, 2 mM L-glutamine (Sigma-Aldrich, G8540), 1% Pen/Strep, 1 mM sodium pyruvate (Sigma-Aldrich, P5280), 1,000 units per mL Leukemia Inhibitory Factor (LIF, Millipore, LIF2005), 1× Minimum Essential Medium Nonessential Amino Acids (MEM NEAA, Invitrogen, 11140050), and 50 μM β-Mercaptoethanol (Thermo Fisher Scientific, cat. no. 21985023). When used, adherent V6.5 mESC were washed twice in 1× PBS, detached using 0.25% Trypsin-EDTA (Thermo Fisher Scientific, cat. no. 25200072) for 5 min, diluted in complete culture medium, collected by centrifuge at 350 × *g* for 5 min, and resuspended in complete medium.

### Sample Dissection and Single-Cell Isolation.

Mouse cortex nuclei were isolated based on the protocol from ref. 55. Briefly, the mouse used in this study was female from the F1 hybrid of male CAST/EiJ (JAX 000928) and female C57BL/6J (JAX 000664) at the age of P42. Cortex was dissected in ice-cold 1× PBS and placed in 1 mL nuclei isolation medium with Triton (0.25 M sucrose, 25 mM KCl, 5 mM MgCl_2_, 10 mM Hepes, pH 8.0, 1 μM DTT, 0.1% Triton X-100) in a 2 mL Dounce homogenizer. Tissues were homogenized with 5 strokes of the loose (A) pestle and 15 strokes of the tight (B) pestle. The homogenate was centrifuged for 8 min at 100 × *g*, 4 °C, and the supernatant was discarded carefully. The pellet was washed twice with 1 mL nuclei isolation medium without Triton (0.25 M sucrose, 25 mM KCl, 5 mM MgCl_2_, 10 mM Hepes, pH 8.0, 1 μM DTT), then filtered with 40 μm strainer.

### METATAC Protocol.

Transposome assembly was performed as previously described (56). The transposase was purified after expression from the pTXB1-Tn5 plasmid (Addgene). Transposon oligonucleotides were synthesized from Invitrogen (polyacrylamide gel electrophoresis [PAGE] purification). META tags used in this study are 16, the same as ref. 57. Each of the transposon strands was dissolved in 0.1× TE to a final concentration of 100 μM. For each of the META tags, two strands were annealed at a final concentration of 5 μM and then pooled with equal volumes. For transposome preparation, equal amounts of Tn5 transposase and the annealed transposon were mixed and incubated for 30 min at room temperature in the dark. Transposome was assembled at a final concentration of 2.5 μM monomer. The resulting assembly was aliquoted and stored at −80 °C until use.

We increased sensitivity from three key modifications. First, for nuclei extraction, we use Omni-ATAC protocol (25), which reduces the mitochondrial reads ratio and increases the signal-to-noise ratio. Second, after transposition, we carry out Tn5 release with an ionic detergent (SDS) treatment, which enabled maximum recovery of DNA yield. Finally, we use META transposome in the transposition step, to avoid half loss compared with the conventional Nextera transposome (24).

Cryopreserved cells were quickly thawed in a 37 °C water bath. Cultured cells or thawed cells were washed twice with ice-cold PBS. Cell number was counted with Hemocytometer (Incyto). A total of 50,000 cells were aliquoted to a 200-μL PCR tube and then pelleted at 500 × *g* for 5 min at 4 °C with a swing bucket centrifuge. Nuclei were extracted with Omni-ATAC protocol (25). Briefly, cells were resuspended in 50 μL ice-cold Omni lysis buffer (10 mM Tris⋅HCl, pH 7.5, 10 mM NaCl, 3 mM MgCl_2_, 0.01% Digitonin, 0.1% Tween-20, 0.1% IGEPAL CA630), pipetted up and down 10 times, incubated on ice for 3 min, washed twice with 100 μL ATAC wash buffer (10 mM Tris, pH 7.5, 10 mM NaCl, 3 mM MgCl_2_, 0.1% Tween-20), and centrifuged at 500 × *g* for 10 min at 4 °C. Nuclei were then resuspended in 25 μL transposition mix (12.5 μL 2× TD buffer from Nextera kit, 10 μL 1× PBS [pH 7.4], 0.25 μL 1% Digitonin, 0.25 μL 10% Tween, 2 μL 1.25 μM META transposome) and incubated on a thermomixer (Eppendorf) at 1,000 rpm, 37 °C for 30 min. The reaction was stopped by adding 25 μL 2× STOP buffer (40 mM EDTA, 10 mM Tris, pH 8.5, 1 mM spermidine) and left on ice for 15 min.

Then we performed plate-based single-cell amplification. All liquid transfer steps were conducted with an acoustic liquid transfer system (Echo 525, Beckman Coulter). Transposed nuclei were resuspended in 1 mL 1× PBS containing 0.5% BSA and sorted to 96-well PCR plates (Eppendorf) containing 1 uL lysis buffer (10 mM Tris, pH 8.0, 20 mM NaCl, 1 mM EDTA, 0.1% SDS, 500 nM Carrier ssDNA, 60 μg/mL QIAGEN protease) by a BD flow cytometer (BD, AriaII). Lysis buffer was aliquot with 384PP_AQ_BP calibration. Lysis buffer could be stored at −80 °C for several weeks. Nuclei were incubated at 65 °C for 15 min to release Tn5 from DNA, then 1 μL 3% Triton X-100 was added to quench SDS. Triton was added with 384PP_AQ_SPHigh calibration. Plates were stored at −80 °C or continued to downstream amplification. Then, 4 μL preamplification mix (3 μL 2× high-fidelity Q5 Master mix, 0.192 μL 50 μM META16 primer mix, 0.05 μL 100 mM MgCl_2_, 0.758 μL H_2_O) were added to each well using 384PP_AQ_BP calibration. META16 primer sequences were in the form of 5′-[META tag]-AGATGTGTATAAG. The preamplification step was performed on Thermocycler with 72 °C, 5 min, 98 °C, 30 s, 16 cycles of [98 °C, 10 s, 62 °C, 30 s, 72 °C, 1 min], 72 °C, 5 min. Then 0.225 μL 50 μM indexed META16-ADP1 primer and 0.225 μL 50 μM META16-ADP2 primer were added to each well with 384PP_AQ_BP calibration. Plates were amplified to incorporate cell barcodes with the following cycle conditions: 98 °C, 30 s, five cycles [98 °C, 10 s, 62 °C, 30 s, 72 °C, 1 min], 72 °C, 5 min. META16-ADP1 primer is in the form of 5′-CTTTCCCTACACGACGCTCTTCCGATCT-NNNNNN (cell barcode)-[META Tag]-AGATGTGTATAAG. META16-ADP2 primers are in the form of 5′-GAGTTCAGACGTGTGCTCTTCCGATCT-NNNNNN (Cell Barcode)-[META Tag]-AGATGTGTATAAG. Finally, a whole plate of PCR products was pooled together and purified with a ZYMO DCC5 kit. All primer sequences are in *SI Appendix*, Table S1.

For library preparation, a unique dual index combination was used to reduce index hopping. Briefly, we took 120 ng purified amplicon from each plate as input, then 21 μL PCR mix (15 μL 2× Q5 Master mix, 3 μL NEBNext index primer i5, and 3 μL NEBNext index primer i7, 0.05 μL 100 mM MgCl_2_) were added and then incubated at 98 °C, 30 s, two cycles of [98 °C, 10 s, 68 °C, 30 s, 72 °C, 1 min], 72 °C, 5 min. Library was purified with ZYMO DCC5 kit, then purified with 1.1× SPRIselect beads to remove the residual primer.

To estimate the collision rate introduced by sample processing steps and flow cytometry, we conducted species mixing experiments. We mixed an equal number of cells from the human cell line (K562) and mouse cell line (mESC V6.5) before processing, then processed together from permeabilization to bulk transposition and sequenced the resulting library at the same sequencing depth.

### Single-Cell Joint ATAC–RNA Assay.

We adopted a modified cell cross-link method for multiomics experiment to retain more cytoplasmic mRNA. For fresh prepared single-cell suspension, wash once with ice-cold PBS, count cell number, aliquot 50,000 cells to a 200-μL PCR tube, then resuspend in 200 μL ice-cold 4% PFA in PBS, incubate on ice for 10 min, then quench by adding 600 μL 1M Tris (pH 7.5). Then wash twice with 200 μL ice-cold PBS containing 1% BSA supplemented with 1 U/μL RNase inhibitor. Snap frozen with liquid nitrogen, then store at −80 °C.

Thaw cell pellet on ice, then permeabilize cells with 50 μL (10 mM Tris⋅HCl, pH 7.5, 10 mM NaCl, 3 mM MgCl_2_, 0.01% Digitonin, 0.1% Tween-20, 0.2% IGEPAL CA630, 1 U/μL RNase inhibitor), incubate on ice for 5 min, then wash twice with 100 μL (10 mM Tris, pH 7.5, 10 mM NaCl, 3 mM MgCl_2_, 0.1% Tween-20, 1 U/μL RNase inhibitor) and centrifuge at 500 × *g* for 5 min at 4 °C. Nuclei were then resuspended in 25 μL transposition mix (12.5 μL 2× TD buffer from Nextera kit, 8.75 μL 1× PBS [pH 7.4], 0.25 μL 1% Digitonin, 0.25 μL 10% Tween, 2 μL 1.25 μM META transposome, 1.25 μL 20 U/μL RNase inhibitor) and incubated on a thermomixer (Eppendorf) at 500 rpm, 37 °C for 30 min. The reaction was stopped by adding 25 μL 2× STOP buffer (40 mM EDTA, 10 mM Tris, pH 8.5, 1 mM spermidine, 1 U/μL RNase inhibitor) and left on ice for 15 min.

Reverse transcription and cDNA second strand synthesis were adopted from MALBAC-DT (23). Briefly, cells were sorted to a 96-well plate, containing 1 μL lysis buffer (1× SSIV buffer, 1 mM dNTP, 5% PEG8000, 0.5% Nonidet P-40, 0.5 U/μL SUPERase inhibitor, 50 μM RT primer “A”), incubated at 72 °C for 10 min, held at 4 °C to make polyT primer binding. Then add 1 μL RT mix (1× SSIV buffer, 5 mM DTT, 6 mM MgSO_4_, 2 U/μL SSIV RTase, 1 U/μL SUPERase inhibitor); reverse transcription incubates at 55 °C for 10 min. For Tn5 release, add 0.25 μL 0.3% SDS, incubate at 65 °C for 15 min, then add 0.25 μL 9% Triton X-100 to quench SDS. To digest excess RT primers and avoid rebinding during the preamplification step, causing overcounting of UMIs, 2 μL EXOI mix (1× EXOI buffer, 2.5 μM primer “B,” 5 U/μL EXOI) were added, incubate at 37 °C, 30 min, 80 °C, 20 min.

For cDNA second strand synthesis, add 10 μL PCR mix (1× Thermopol Buffer, 0.2 mM dNTP, 1.33 mM MgSO_4_, 0.67 μM GAT5-B1-7N, 0.67 μM GAT5-B1-Com, 0.1 U/μL Deep Vent [exo-]). Then incubate at 72 °C, 5 min; 95 °C, 5 min; 11 cycles of [4 °C, 50 s, 10 °C, 50 s, 20 °C, 50 s, 30 °C, 50 s, 40 °C, 45 s, 50 °C, 45 s, 65 °C, 4 min, 95 °C, 20 s, 58 °C, 20 s].

After cDNA second strand synthesis, we performed ATAC fragment and cDNA preamplification, then split as two parts for cDNA and DNA enrichment, respectively. Add 0.5 μL 50 μM META16 primer mix, incubate at 95 °C, 1 min; five cycles of [95 °C, 20 s, 58 °C, 30 s, 72 °C, 3 min], 72 °C, 5 min. Then split as two parts, 5 μL for ATAC DNA enrichment, by adding 5 μL DNA enrichment mix (1× Thermopol Buffer, 0.2 mM dNTP, 1.6 mM MgCl_2_, 3.2 μM META16 primer mix, 0.2 U/μL Deep Vent [exo-]), incubate at 95 °C, 1 min; 12 cycles of [95 °C, 20 s, 62 °C, 30 s, 72 °C, 1 min], 72 °C, 5 min, then add 0.65 μL META16-ADP1-ADP2 cell barcode to each well, incubate at 95 °C, 1 min; five cycles of [95 °C, 20 s, 62 °C, 30 s, 72 °C, 1 min], 72 °C, 5 min. For cDNA enrichment, take 10 μL preamplification product, add 10 μL cDNA enrichment mix (1× Thermopol Buffer, 0.2 mM dNTP, 1.6 mM MgCl_2_, 1 μM GAT5-B1-Com, 0.2 U/μL Deep Vent [exo-]), incubate at 95 °C, 1 min; 12 cycles of [95 °C, 20 s, 58 °C, 30 s, 72 °C, 3 min], 72 °C, 5 min. Then add 0.5 μL 20 μM primerG to add Illumine sequencing adaptor to 3′, incubate at 95 °C, 1 min; five cycles of [95 °C, 20 s, 58 °C, 30 s, 72 °C, 3 min], 72 °C, 5 min. After amplification, pool a whole plate for purification.

For cDNA library preparation, we take 50 ng (2 μL) as input for each plate, then tagment with Nextera transposome by adding 6 μL transposition mix (leading to a final concentration of 10 mM TAPS, pH 8.5, 5 mM MgCl_2_, 8% PEG 8000, 28 nM Nextera transposome dimer), incubation at 55 °C for 10 min. Then add 2 μL stop mix (250 mM NaCl, 37.5 mM EDTA, 2 mg/mL QIAGEN protease) and incubation at 50 °C for 30 min, 70 °C for 15 min. Add 20 μL PCR mix (15 μL 2× Q5 master mix, 0.5 100 mM MgCl_2_, 2 μL i5 index, 2 μL i7 index), incubate at 72 °C, 5 min, 98 °C, 30 s, 12 cycles of [98 °C, 10 s, 62 °C, 30 s, 72 °C, 1 min], 72 °C, 5 min. Then size selection with first 0.55×, then 0.25× SPRIselect beads. All MALBAC-DT–related oligos sequences are the same as ref. 28 and could be found in *SI Appendix*, Table S1.

### Sequencing.

METATAC library were sequenced with paired-end 2 × 150 bp on an Illumina Hiseq 4000/Hiseq ×10; 10% PhiX (Illumina; FC-110-3001) were added to avoid low complexity at 19-bp mosaic end sequence. Typically, each single cell was sequenced to 500,000 reads, 15G base/96-well plate. MALBAC-DT library was sequenced with paired-end 2 × 150 bp on an Illumina Hiseq 4000/Hiseq ×10. Typically, each single cell was sequenced to 500,000 to 1 million reads, 15 to 30G base/96-well plate.

### Raw Read Preprocessing.

For both read 1 and read 2, the first 4 to 7 bases and the following 11 to 13 bases are paired cell barcodes and META sequence, respectively (*SI Appendix*, Table S1). We used a custom Python script to parse barcodes and split reads into individual fastq files for each cell, allowing up to one mismatch. Meanwhile, META sequences were annotated to read the name, allowing up to two mismatches. Then we used cutadapt to trim adapter sequences from both ends according to the 19-bp mosaic end (ME) sequence, with parameters -e 0.22 -a CTGTCTCTTATACACATCT and -e 0.22 -g AGATGTGTATAAGAGACAG for both read 1 and read 2. Processed reads were mapped to reference genome with bowtie2 -X 2000 –local –mm –no-discordant –no-mixed. hg38 (GRCh38, v26) reference genome was used for human cells, and mm10 (GRCm38, vM19) reference genome was used for mouse cells. Reads with mapping quality less than 30 were filtered out from further analysis. PCR duplicates were identified and removed with a custom script, according to their positions on genome and META tags. Paired-end reads were converted to fragments with Tn5 insertion centering correction (R1 start +4 and R2 end −5). Finally, for each cell, contaminated fragments from other cells were removed based on the aligned coordinates, META sequences, and read frequency.

### QC for METATAC.

The preprocessed fragments of all cells were input to ArchR (Version 0.9.4) (34) to create an ArchR object. Cells were filtered based on several criteria: number of reads, alignment rate, number of fragments, contamination rate, mitochondrial fragment rate, TSS enrichment score, promoter ratio, and doublet enrichments. About 10% of cells would be filtered (*SI Appendix*, Table S3).

### Cell Clustering and Peak Calling.

After cell filtering, iterative latent semantic indexing was performed using ArchR to reduce dimensions (13, 58). Cells were clustered using Seurat’s FindClusters function (59) and embedded using the addUMAP function. The marker genes were identified by the getMarkerFeatures function with gene score matrix calculated by ArchR. Cell clusters were annotated based on the marker gene expressions (33). Chromatin accessible peaks were called by addReproduciblePeakSet function using MACS2 (60). The length of each peak was fixed as 501 bp.

### Species Mixing Experiment.

The sequencing reads were aligned to a combined reference genome of hg38 and mm10. The low-quality cells were filtered out by the number of reads, alignment rate, number of fragments, contamination rate, and mitochondrial fragment rate. For the remaining cells, we calculated the fraction of fragments that aligned to the human chromosomes. If the fraction is larger than 0.9, the cell is classified as a human cell; if the fraction is smaller than 0.1, the cell is classified as a mouse cell. Otherwise, the cell is classified as a doublet.

### Comparison with Previous Methods.

To compare METATAC and other reported scATAC-seq approaches, raw sequencing data of GM12878 and K562 cells from bulk Omni-ATAC-seq, sci-ATAC-seq (Cusanovich), Fluidigm C1, and dscATAC-seq are downloaded from the Sequence Read Archive (SRA) database and processed with a similar pipeline as described above. GM12878 and K562 DNase-seq peak files are downloaded from the ENCODE project (ENCFF962MMN and ENCFF185XRG). After peak calling with MACS2, we count the fragments which intersect with at least one peak as the number of fragments in peaks using bedtools for each cell. The fraction of fragments in DNase-seq peaks is calculated similarly. For scATAC-seq (14), raw sequencing data (fastq files) of K562 cells were downloaded from https://www.ebi.ac.uk/biostudies/arrayexpress/studies/E-MTAB-6714. Nextera adapter sequences were trimmed using cutadapt (v4.0), and then reads were mapped to GRCh38 reference genome. The library complexity was estimated from the mapped BAM file of each cell using Picard EstimateLibraryComplexity (v2.27.3). Mapped fragments of all cells were merged, and peaks were identified using macs2 (v2.2.7.1, with parameters –nomodel –nolambda -B –SPMR –keep-dup all). Number of fragments in peaks were calculated using bedtools intersect (v2.30.0, with parameters -a $FRAGMENT -b $PEAKS). For 10× scATAC-seq, metadata and BAM files containing mapped reads were downloaded from https://www.10xgenomics.com/resources/datasets/10k-1-1-11mixture-of-human-gm12878-and-mouse-el4-cells-atac-v2-chromium-controller-2-standard. Reads from each GM12878 cell (according to the annotation in metadata) were extracted from the BAM file and split into individual BAM files. Then BAM files were processed as processing data in ref. 14. For s3ATAC, metadata of human cortex dataset were downloaded from Gene Expression Omnibus (GEO) (GSM5289636), and library complexities were estimated manually using the Lander–Waterman equation from the “total_reads” and “uniq_reads” columns in the metadata table. For HyDropATAC, fragments were downloaded from GEO (GSM5343842). Number of total fragments, mitochondrial fragments, and unique fragments of each cell were then calculated, and library sizes were estimated as mentioned above.

To investigate correlations among different methods across the two cell lines, we merge the DNase-seq peaks of GM12878 and K562 cells, and all cells are aggregated for single-cell datasets. We count the number of fragments in each peak for each method and cell type and then calculate Spearman’s correlation in pairs. For cell down-sample analysis, we randomly sample a certain number of cells from GM12878 datasets of each method. Fragments of the selected cells are aggregated and served to MACS2 for peak calling. We calculate the number of DNase-seq peaks that intersect with at least one of the resulting down-sample peaks. We perform 10 independent down-sample processes for each method and each cell number, to reduce random noise and give a standard derivation as error bars (Fig. 1*G*).

### Coaccessibility Score.

The coaccessibility score of a pair of chromatin regions was defined by a hypergeometric test. Suppose the number of cells was N. If region 1 was detected to be accessible within M cells and region 2 was detected to be accessible within n cells, then if the accessibilities of these two regions are independent with each other, the number of cells with both regions accessible, denoted as X, is a random variable that follows a hypergeometric distribution. If these two regions were detected to be both accessible within x cells, then the coaccessible score was computed as follows:

$$\mathrm{score}=-\log_{10}\left(P\left(X\geq x\right)\right)=-\log_{10}\left(\underset{k\geq x}{\sum }\frac{C_{M}^{k}C_{N-M}^{n-k}}{C_{N}^{n}}\right),$$

where C represents the number of combinations. This score was a nonnegative value. A high score indicated the two regions are highly coaccessible.

### Monoallelic Accessibility.

The allele-specific alignment step followed ref. 46. First, SNP sites between CAST/EiJ and the reference genome (C57BL/6J) were downloaded from Mouse Genomes Project (REL-1505) (37, 61). The SNP sites were replaced by “N” in the reference genome. Then, processed reads were aligned to the modified reference genome to remove mapping bias. Reads aligned to SNP sites were labeled as paternal or maternal according to the genotype. Reads not aligned to SNP sites, those that contained SNP of both parents, or those in which the genotype was inconsistent with either parent are labeled as inconclusive and were excluded for allele-specific analysis. Finally, for each accessible peak, the monoallelic accessibility was calculated by the number of inserts from corresponding parental fragments.

### Allele Specificity.

The quantification of allele specificity was based on a binomial test. For a given peak, assume its paternal accessibility was n, while its maternal accessibility was m. If both alleles are equally accessible, then the paternal accessibility is a random variable that follows binomial distribution, with a probability of 0.5. The allele specificity was computed as follows:

$$S=\log_{10}\left(\overset{n+m}{\underset{k=n}{\sum }}C_{n+m}^{k}{\left(\frac{1}{2}\right)}^{k}\right)-\log_{10}\left(\overset{n+m}{\underset{k=m}{\sum }}C_{n+m}^{k}{\left(\frac{1}{2}\right)}^{k}\right).$$

A positive S indicates the maternal allele is more accessible, and vice versa.

### Joint ATAC–RNA Methods Benchmark.

For simultaneous METATAC and MALBAC-DT profiling benchmark, we downloaded the processed count matrices or fragments of different cell lines from other methods from GEO, including Paired-Seq, simultaneous high-throughput ATAC and RNA expression with sequencing (SHARE-seq), single-nucleus chromatin accessibility and mRNA expression sequencing (SNARE-seq), and single-cell combinatorial indexing joint chromatin accessibility and mRNA (sciCAR). The per-cell number of UMIs or fragments in peaks are calculated from the count matrix, by summing the count values across all genome regions for ATAC or genes for RNA-seq. For 10× ATAC-RNA multiomics, metadata of the human peripheral blood mononuclear cell (PBMC) dataset were downloaded from https://www.10xgenomics.com/resources/datasets/pbmc-from-a-healthy-donor-granulocytes-removed-through-cell-sorting-3-k-1-standard-1-0-0. For assay for single-cell transcriptome and accessibility regions (ASTAR-seq), K562 ATAC raw sequencing reads were downloaded from SRA (SRP265830) and processed as described above. For RNA-seq comparison, count matrix was downloaded from GEO (GSE113415). For SNuBar-ARC, count matrices of both ATAC and RNA parts were downloaded from GEO (GSM4960042 and GSM5494071).

### Raw Reads Preprocessing for Joint ATAC–RNA.

The raw reads preprocessing for ATAC-seq library was the same as that for METATAC. For RNA-seq library, we used the pipeline developed for MALBAC-DT (23). First, the raw reads for each 96-well plate were demultiplexed based on the cell barcodes in the R2 reads, where at most one mismatch was allowed. The demultiplexed R2 reads which contained more than three bases inconsistent to designed UMI patterns or contained less than 4 T in the 5 bp downstream regions of UMIs were filtered. For the remaining R1 reads, the polyA sequences were trimmed, followed by filtering for high-quality reads with the following criteria: 1) not less than 40 bp, 2) more than half of the bases have sequence quality scores greater than 38, and 3) less than 10% of bases are N.

The processed R1 reads were mapped to the hg38 or mm10 genome using Spliced Transcripts Alignment to a Reference (STAR) (2.5.3a) (62). The uniquely mapped reads were kept and assigned to genes using htseq-count in HTSeq (0.11.2) (63). For each gene, the UMIs with a hamming distance of no more than 1 were collapsed. Finally, the gene expression matrix was output using the UMI count for each gene.

### QC for Joint ATAC–RNA.

The QC for the ATAC-seq data was the same as that for METATAC data. For RNA-seq data, cells were filtered based on several criteria: ratio of reads with correct UMI pattern, number of reads after filtering, alignment rate, detected gene number, detected UMI number, mitochondrial gene UMI rate, and External RNA Controls Consortium (ERCC) rate (*SI Appendix*, Table S3). Cells passing QC for both RNA-seq and ATAC-seq were kept for further analyses.

## Supplementary Material

Supplementary File

## Data Availability

Raw sequencing data are available at the SRA (accession no. PRJNA789047 [64]). Code is available via GitHub (https://github.com/sunneyxielab/METATAC_pipeline).
