# Supplementary material for: Highly sensitive single-cell chromatin accessibility assay and transcriptome coassay with METATAC
Source: Proc Natl Acad Sci U S A. 2022 Sep 26;119(40):e2206450119. doi: 10.1073/pnas.2206450119 (PMC9546615; doi:10.1073/pnas.2206450119)
Supplement: Supplementary File [file pnas.2206450119.sapp.pdf]

PNAS
www.pnas.org

Supplementary Information for
Highly sensitive single-cell chromatin accessibility assay and transcriptome co-assay with METATAC

Honggui Wu[a, b, c, 1], Xiang Li[a, b, d, 1, 2], Fanchong Jian[a, b, e, 1], Ayijiang Yisimayi[a, b, c, 1], Yinghui Zheng[a, b], Longzhi Tan[f], Dong Xing[a, b], X. Sunney Xie[a, b, c, d, *]

[a]Biomedical Pioneering Innovation Center (BIOPIC), Peking University, Beijing, 100871, China

[b]Beijing Advanced Innovation Center for Genomics (ICG), Peking University, Beijing, 100871, China

[c]School of Life Sciences, Peking University, Beijing, 100871, China

[d]Peking-Tsinghua Center for Life Sciences (CLS), Academy for Advanced Interdisciplinary Studies, Peking University, Beijing, 100871, China

[e]College of Chemistry and Molecular Engineering, Peking University, Beijing, 100871, China

[f]Department of Bioengineering, Stanford University, Stanford, CA 94305, USA

[1]These authors contributed equally

[2]Present address: Department of Statistics and Actuarial Science, The University of Hong Kong, Pokfulam Road, Hong Kong

*X. Sunney Xie
Email: sunneyxie@biopic.pku.edu.cn

This PDF file includes:

Figures S1 to S9
SI References

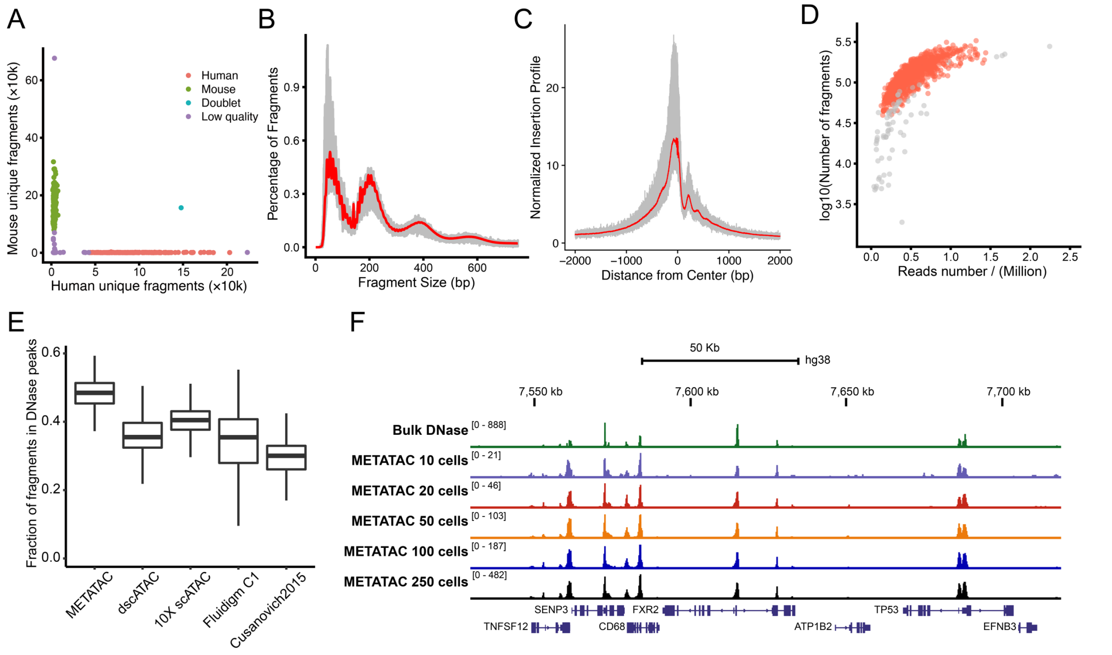

Fig. S1. METATAC shows high quality in GM12878. (*A*) Species mixing experiments to show the low cell-cell contamination and FACS sorting accuracy. Equal numbers of mESC v6.5 (mouse) and K562 (human) cells are mixed before nuclei extraction. (*B*) Fragment size distribution for METATAC, grey line represents 10 randomly selected single cells, red line shows merged single cell data. (*C*) Insertion frequency around TSSs regions, TSSs are enriched nucleosome-free fragments. (*D*) sequencing depth versus detected unique fragments number in a single cell. (*E*) Fraction of mapped reads in DNaseI hypersensitive sites across five technologies for GM12878 cells, the median for METATAC is 48.5%, as compared to dscATAC (1) (35.5%), 10x scATAC (2) (40.5%), Fluidigm C1 (3) (35.5%), sciATAC-seq methods (4) (30%). (*F*) Comparison of the aggregate chromatin accessibility profiles from GM12878 cells using different cell numbers, downsample was randomly performed 4 times for each cell number.

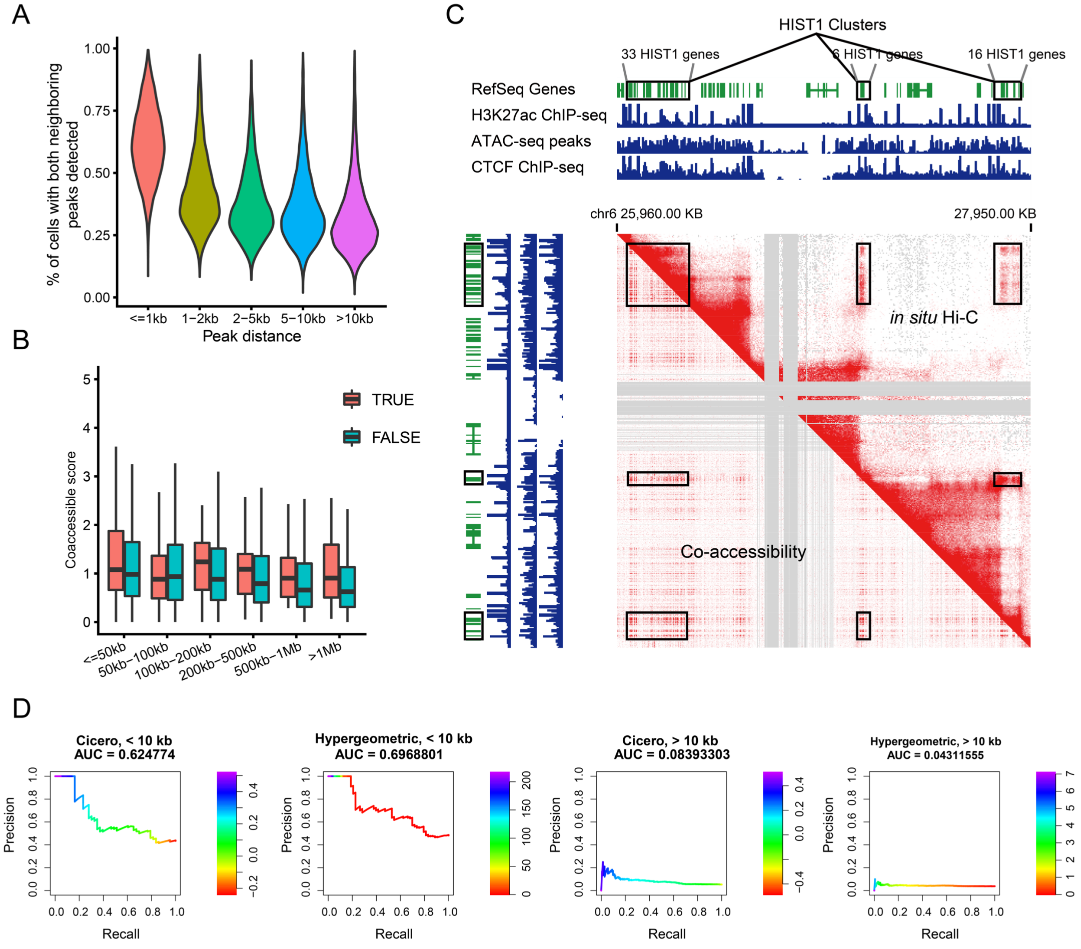

Fig. S2. Co-accessibility of functional elements. (*A*) As in Fig. 2*B*, calculated by the percentage of both peaks detected cells. (*B*) Co-accessibility scores for a CRISPRi datasets (5), grouped by distance, which was calculated with K562 cells. (*C*) Hi-C contact map and co-accessibility around histone gene cluster 1 on chromosome 6 (known interactions between clusters are labeled). (*D*) Precision-recall curve of hypergeometric test and Cicero, calculated from CRISPRi dataset (5), E-P pairs within 10 kb and > 10 kb were calculated separately

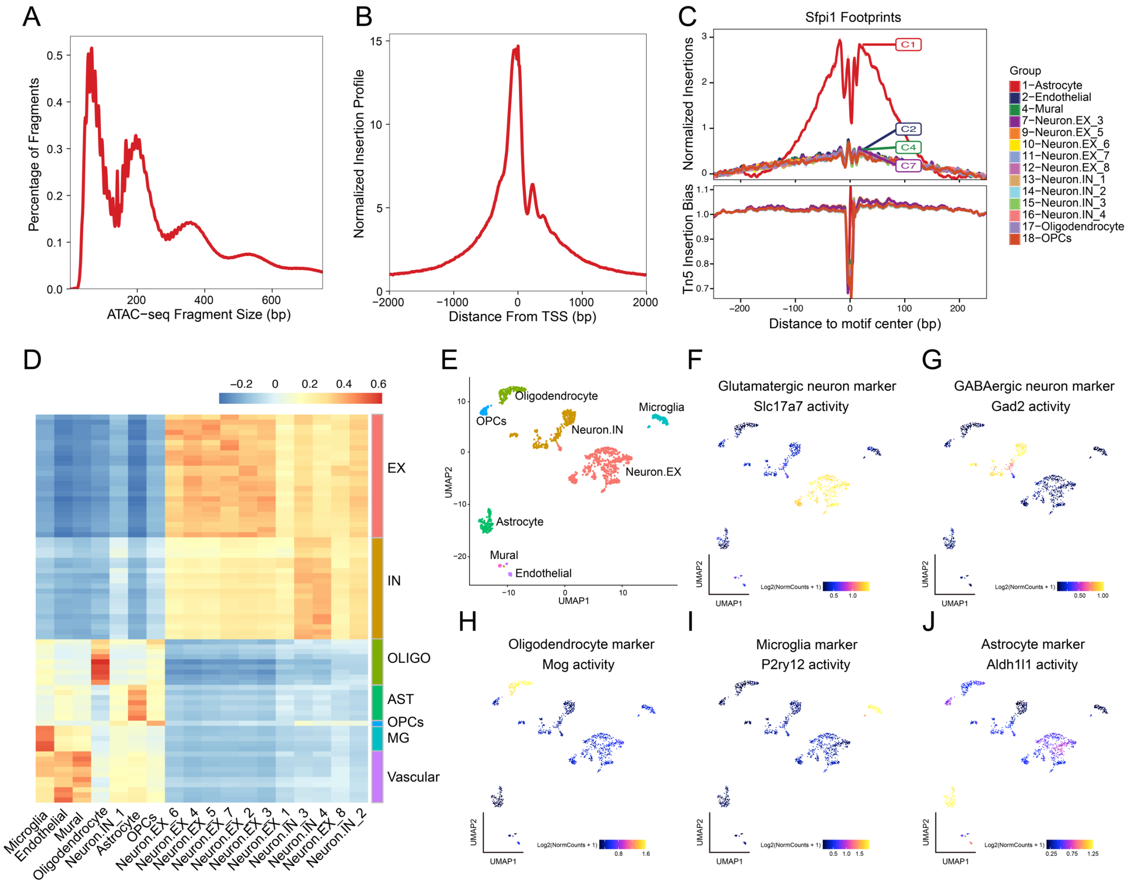

Fig. S3. Quality control and cell type annotation of mouse cerebral cortex. (*A*) Fragment size distribution. **(***B*) Tn5 insertion profile around TSS. (*C*) TF footprints of the Sfpi1 motif. (*D*) Correlation matrix between clusters defined by scRNA-seq (6) and METATAC. Spearman correlation was calculated by all genes. (*E*) UMAP of mouse cerebral cortex, colored by major cell types. (*F-J*) Gene activity of known marker genes, including Slc17a7(*F*; Glutamatergic neuron), Gad2 (*G*; GABAergic neuron), Mog (*H*; oligodendrocyte), P2ry12 (*I*; microglia), and Aldh1l1 (*J*; astrocyte).

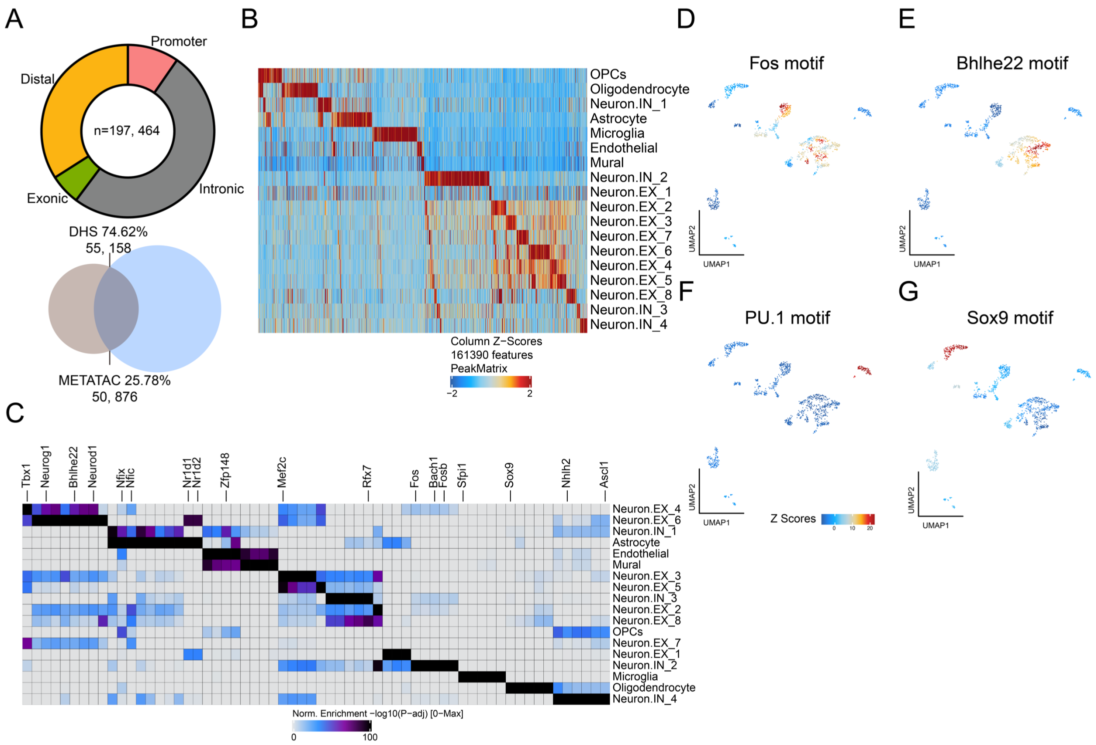

Fig. S4. Cell-type-specific epigenomic features in mouse cerebral cortex. (*A*) Fraction of identified cCREs overlapped with TSS, intron, exon and intergenic regions, Venn diagram indicating overlap between cCREs and DNaseI hypersensitive sites (DHSs) from mouse brain cortex. (*B*) Chromatin accessibility signal of 161,390 cell-type-specific peaks of mouse brain cerebral cortex. (*C*) Enriched TFs for each cell type. (*D-G*) Cell-type-specific activity of known TF regulators, plots depict the chromVAR deviation score, including for Fos (*D*; reflecting neural activity), Bhlhe22 (*E*; excitatory neuron), PU.1 (*F*; microglia), and Sox9 (*G*; oligodendrocyte).

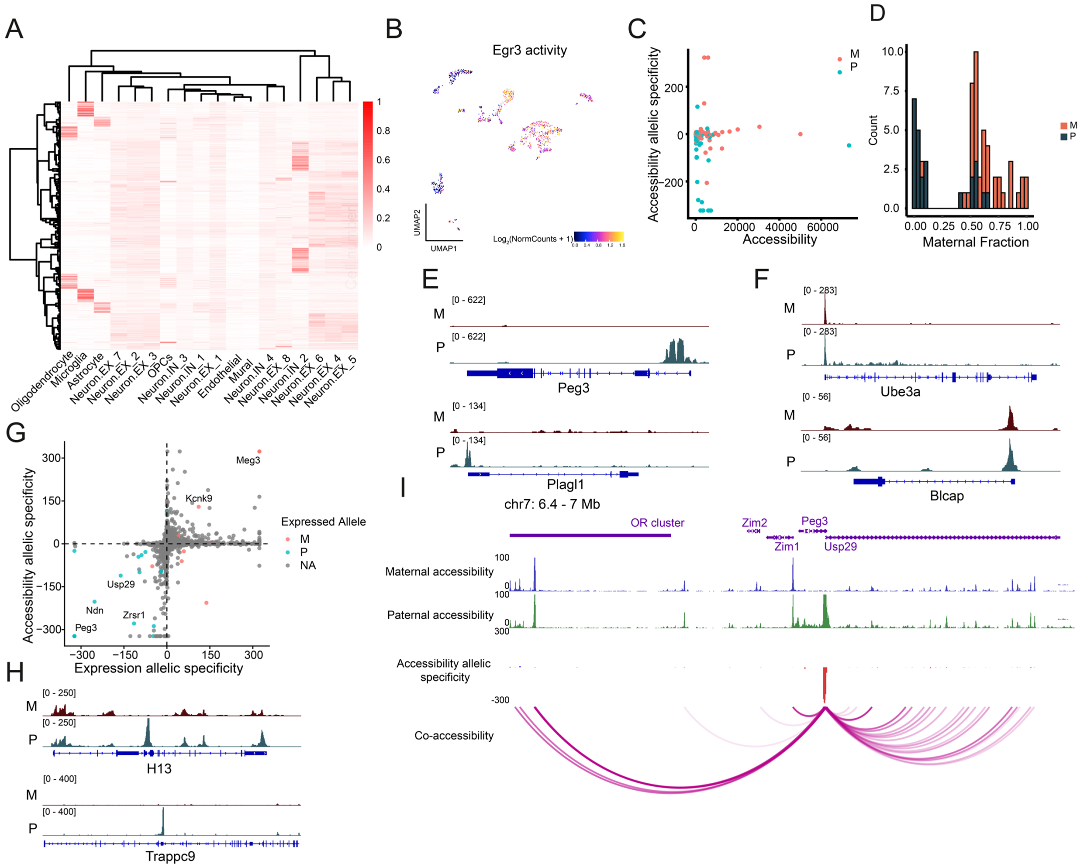

Fig. S5. Allele-specific accessibility of mouse cerebral cortex. (*A*) Corresponding to Fig. 3*B*, colored by accessibility. (*B*) Cells are colored by Egr3 gene activity. (*C*) scatter plot of accessibility level versus accessibility allelic specificity. (*D*) Maternal expression fraction of all the detected imprinted genes. (*E*) Aggregated scATAC-seq tracks for represented paternally imprinted genes (*F*) Aggregated scATAC-seq tracks for represented maternally imprinted genes. (*G*) Scatter plot of accessibility allele specificity versus gene expression allele specificity for all bulk RNA-seq detected genes. (*H*) Aggregated scATAC-seq tracks for represented isoform specific imprinting genes in Neuron.EX6. (*I*) Example of linked enhancers of imprinted genes, genome track shows Peg3 and Usp29 region, tracks including maternal accessibility, paternal accessibility and accessibility specificity, and co-accessibility are shown.

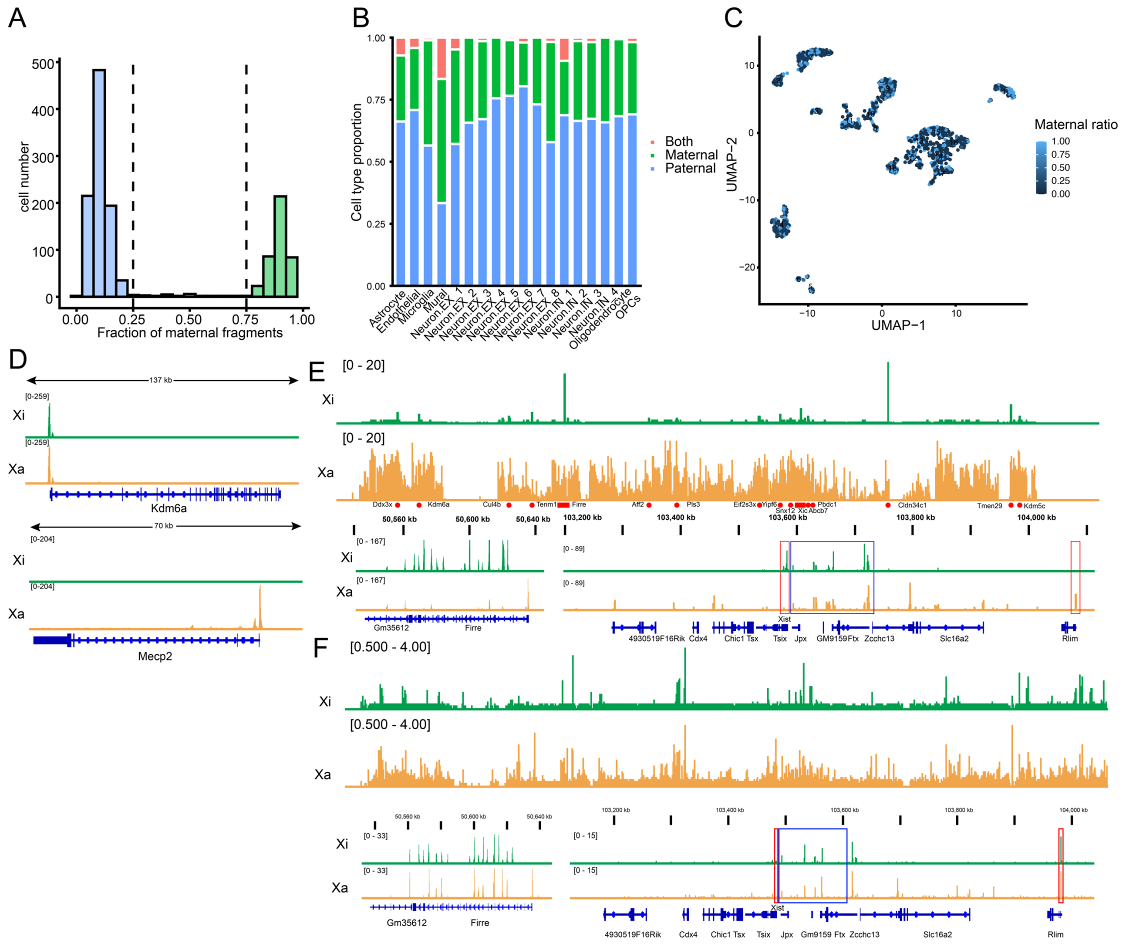

Fig. S6. X chromosome accessibility landscape in mouse cerebral cortex. (*A*) Fraction of maternal fragments in all SNP-informative fragments for X chromosome in single cells, near 0 indicates Xm inactivation, near 1 indicates Xp inactivation. (*B*) Fraction of Xp/Xm inactivation in each cell type. (*C*) cells are colored by X chromosome maternal ratio. (*D*) Exemplified escapees accessibility, upper brain escapee, lower non-brain escapee. (*E*) Upper: active and inactive X chromosome accessibility landscape for mouse cerebral cortex, lower: exemplified region accessibility, left, Firre locus, right, X inactivation center. (*F*) The same as (*E*) for neuron progenitor cells, data from (7).

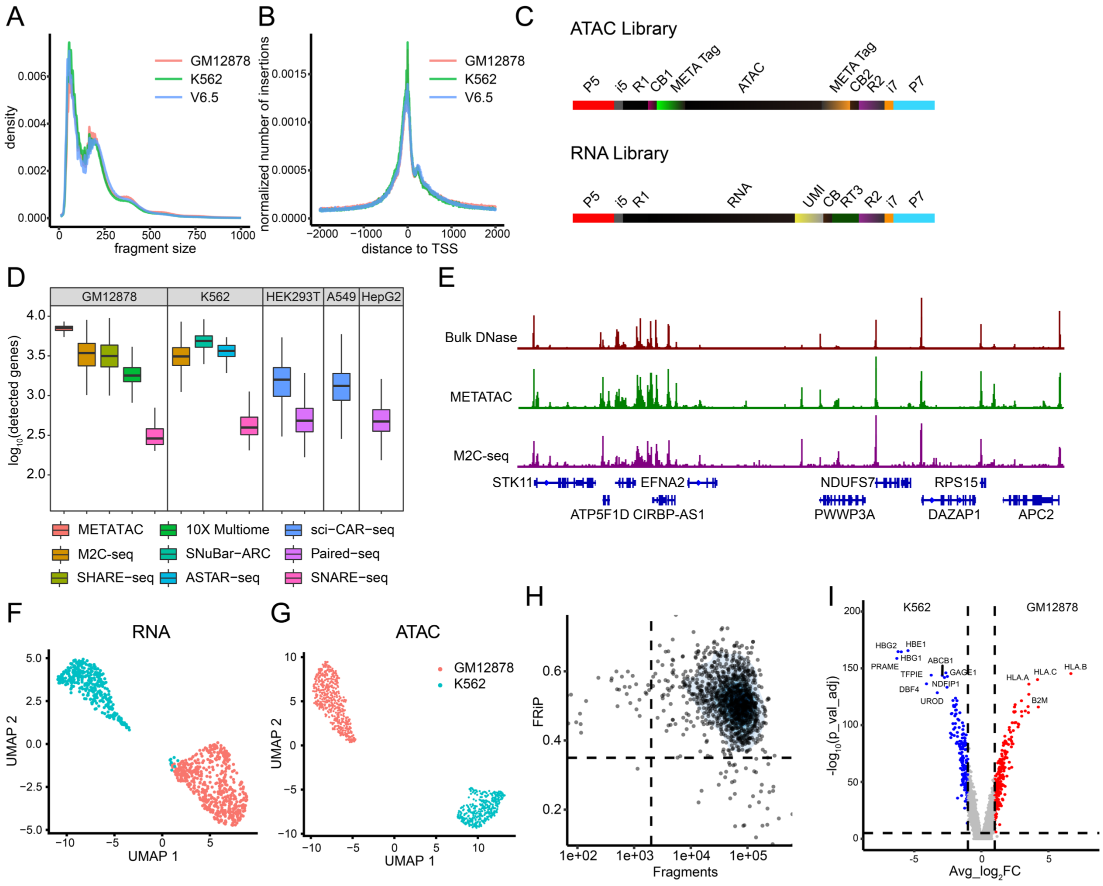

Fig. S7. Data quality control of our joint method on cell line datasets. (*A*) The insert size distribution of ATAC-seq fragments. (*B*) Enrichment of ATAC-seq fragments around TSS. (*C*) depiction of ATAC and RNA library structure. (*D*) Detected gene number for cell lines, the median number of M2C-seq is (GM12878, 3,438, n = 540; K562, 3,113, n = 379), as compared to MALBAC-DT only (GM12878, 7,092, n = 948), SHARE-seq (GM12878, 3,151, n = 1,125), 10X multiome (GM12878, 1,791, n = 2,714), SNuBar-ARC (K562, 4,870, n = 5,825), ASTAR-seq (K562, 3,654, n = 192), sci-CAR (HEK293T, 1,588, n = 812; A549, 1,324, n = 4,277), SNARE-seq (GM12878, 288, n = 140; K562, 396, n = 200), Paired-seq (HEK293T, 483, n = 1,174; HepG2, 472, n = 1,141). (*E*) Aggregate scATAC-seq tracks in GM12878 cells, METATAC, and bulk DNase are shown. (*F*) UMAP clustering of GM12878 and K572, defined by RNA. (*G*) UMAP clustering of GM12878 and K572, defined by ATAC. (*H*) Scatterplot of the fraction of reads in peaks for GM12878 cell line. (*I*) Volcano plot of differentially expressed genes between GM12878 and K562.

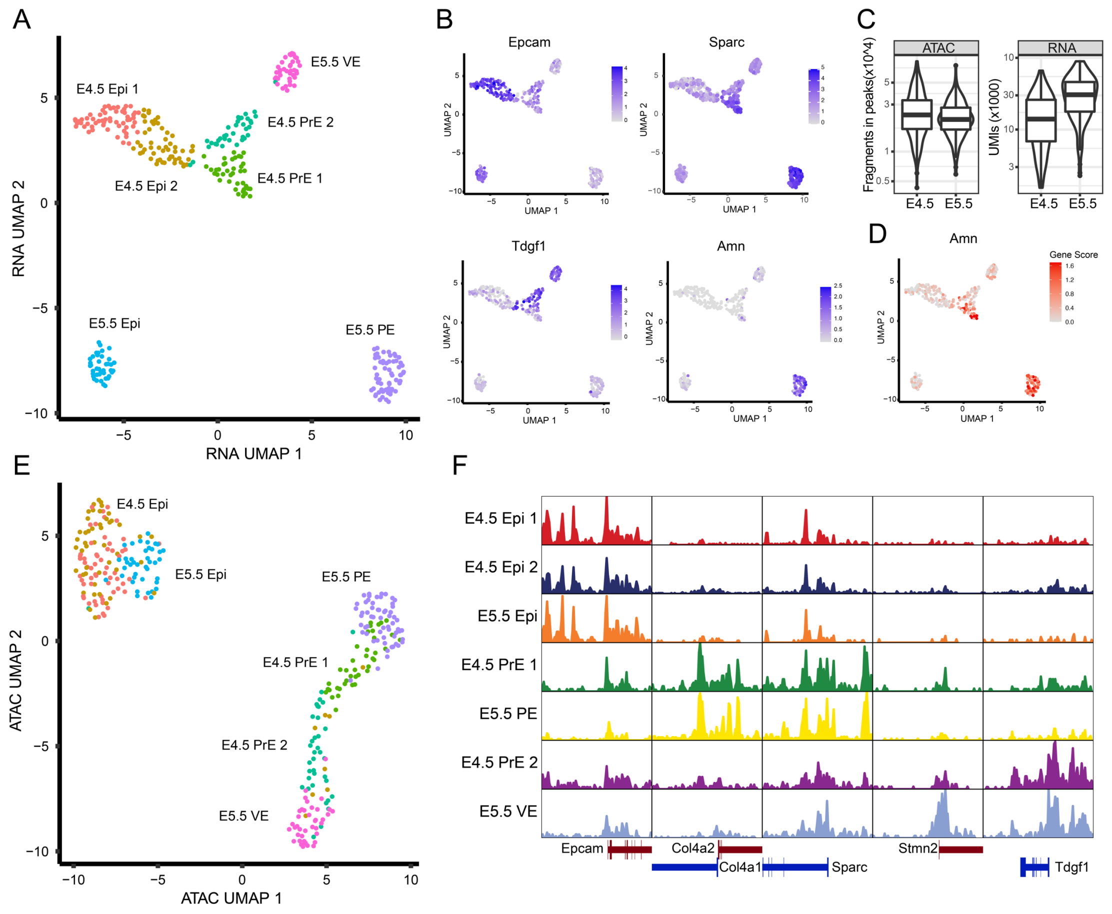

Fig. S8. Joint profiling of transcriptome and chromatin accessibility in E4.5-E5.5 mouse embryo. (*A*) UMAP visualization of single cells from mouse embryo showing UMAP coordinates defined by RNA. Cluster colors are defined by RNA clustering. (*B*) cells are colored by the expression of marker genes (Epcam, Sparc, Tdgf1, and Amn). (*C*) Fragments in peaks and UMIs of E4.5 and E5.5 cells. (*D*) cells are colored by the gene activity of Amn. (*E*) UMAP visualization of single cells from mouse embryo showing UMAP coordinates defined by ATAC. Cluster colors are defined by RNA clustering. (*F*) Aggregated scATAC-seq tracks denoting marker chromatin accessibility peaks for each cluster.

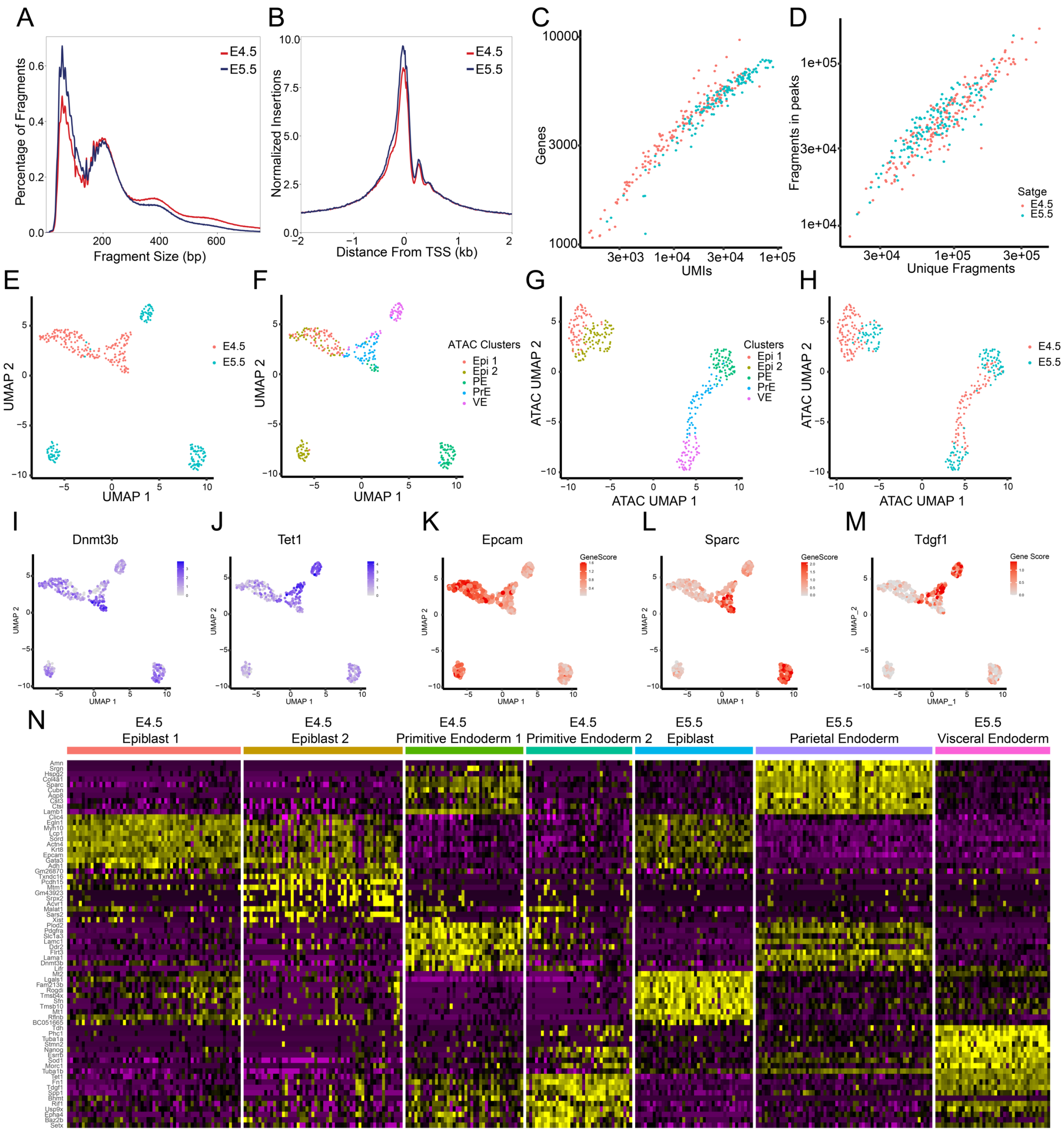

Fig. S9. High-quality data of mouse embryo. (*A*) The insert size distribution of ATAC-seq fragments. (*B*) Enrichment of ATAC-seq fragments around TSS. (*C*) Scatterplot of fragments in peaks. (*D*) Scatterplot of gene number and UMIs. (*E*) RNA UMAP plot, cells are colored by embryo stage. (*F*) RNA UMAP plot, cells are colored by ATAC clusters. (*G*) ATAC UMAP plot, cells are colored by ATAC cell types. (*H*) ATAC UMAP plot, cells are colored by embryo stage. (*I*-*J*) UMPA projection of gene expression for Dnmt3b (*I*) and Tet1 (*J*). (*K*-*M*) UMAP projection of gene activity from scATAC-seq, Epcam (*K*), Sparc (*L*), and Tdgf1 (*M*). (*N*) Heatmap showing expression of markers for each cell type.

SI References

1. C. A. Lareau *et al.*, Droplet-based combinatorial indexing for massive-scale single-cell chromatin accessibility. *Nat Biotechnol* **37**, 916-924 (2019).
2. A. T. Satpathy *et al.*, Massively parallel single-cell chromatin landscapes of human immune cell development and intratumoral T cell exhaustion. *Nature Biotechnology* **37**, 925-936 (2019).
3. J. D. Buenrostro *et al.*, Single-cell chromatin accessibility reveals principles of regulatory variation. *Nature* **523**, 486-490 (2015).
4. D. A. Cusanovich *et al.*, Multiplex single cell profiling of chromatin accessibility by combinatorial cellular indexing. *Science* **348**, 910-914 (2015).
5. C. P. Fulco *et al.*, Activity-by-contact model of enhancer-promoter regulation from thousands of CRISPR perturbations. *Nat Genet* **51**, 1664-1669 (2019).
6. A. Saunders *et al.*, Molecular Diversity and Specializations among the Cells of the Adult Mouse Brain. *Cell* **174**, 1015-1030.e1016 (2018).
7. J. Xu *et al.*, Landscape of monoallelic DNA accessibility in mouse embryonic stem cells and neural progenitor cells. *Nature Genetics* **49**, 377-386 (2017).
